# Considerations for Determining the Coefficient of Inertia Masses for a Tracked Vehicle

**DOI:** 10.3390/s20195587

**Published:** 2020-09-29

**Authors:** Octavian Alexa, Iulian Coropețchi, Alexandru Vasile, Ionica Oncioiu, Lucian Ștefăniță Grigore

**Affiliations:** 1 Military Technical Academy “FERDINAND I”, 39-49 George Coșbuc Av., 050141 Bucharest, Romania; alexa.octavian@gmail.com (O.A.); iulian.coro@gmail.com (I.C.); alexandru.vasile@mta.ro (A.V.); lucian.grigore64@gmail.com (L.Ș.G.); 2 Faculty of Finance-Banking, Accountancy and Business Administration, Titu Maiorescu University, 040051 Bucharest, Romania

**Keywords:** tracked vehicle, mobility, stability, acceleration, torque converter, engine, coefficient of inertia masses, hydro-clutches

## Abstract

The purpose of the article is to present a point of view on determining the mass moment of inertia coefficient of a tracked vehicle. This coefficient is very useful to be able to estimate the performance of a tracked vehicle, including slips in the converter. Determining vehicle acceleration plays an important role in assessing vehicle mobility. Additionally, during the transition from the Hydroconverter to the hydro-clutch regime, these estimations become quite difficult due to the complexity of the propulsion aggregate (engine and hydrodynamic transmission) and rolling equipment. The algorithm for determining performance is focused on estimating acceleration performance. To validate the proposed model, tests were performed to determine the equivalent reduced moments of inertia at the drive wheel (gravitational method) and the main components (three-wire pendulum method). The dynamic performances determined during the starting process are necessary for the validation of the general model for simulating the longitudinal dynamics of the vehicle. Finally, the differential and algebraic equations of the virtual model approximate more accurately the actual process of the operation of the vehicle. The virtual model, through the data obtained from the simulation process, allows for the determination, indirectly, of the variation of the mass moment of inertia coefficient and its expression of approximation.

## 1. Introduction

The acceleration resistance generated by the inertial forces during the starting process of the vehicle depends not only on its weight but also on the rotating masses, from the traction engine elements to the vehicle tracks [1]. According to the relation of the moments of inertia *δ* [-] [2], the resistance generated by the inertia of the rotating elements depends not only on the mass but also on their radius. In the literature [3,4,5], the effect generated by the moving masses of translation and rotation is materialized in the form of the mass moment of inertia coefficient *δ* [6].

The mobility and stability of a vehicle on wheels or tracks are influenced by the moment of inertia [7] but also by the place (on the ground) where gravity acts [8,9]. In [10] is presented a database of the National Highway Traffic Safety Administration (NHTSA), which presents a methodology for calculating not only moments of inertia and center of gravity but also the physical characteristics of the vehicle.

The development of the virtual simulation model aimed at modeling the main parts of a tracked vehicle, such as the 8 V engine, the hydromechanical transmission, the final transmission, and the tracks [10,11,12]. The Matlab programming environment—SIMULINK and SIMSCAPE modules—was used to develop the general simulation model. The simulation model, which refers to the determination of the power flow, is built modularly, and the data on the characteristics of the physical components, used for the construction of the hydromechanical transmission, are found in [13,14]. The modular-type simulation model was generated due to the fact that it will be able to be modified later, depending on the characteristics of the respective components, which come from other equipment manufacturers.

**Figure 1:**
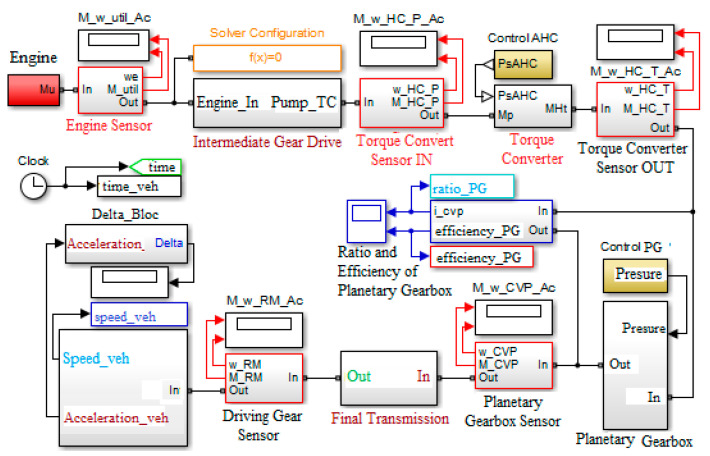
General virtual simulating model of the tracked vehicle [1].

The virtual simulation model has as input elements the experimental data provided by the manufacturer, the main mass and dimensional characteristics of the vehicle, gearshift times, obtained experimentally after experimental determination of the pressure variation in the hydraulic control system at changing gears [3], and the position of the accelerator pedal. The output elements of the virtual model, generated after running the program (simulation process), show the variation over time of the main dynamic characteristics of the tracked vehicle and of the mass moment of inertia coefficient, and are shown in Figure 1.

**Figure 2:**
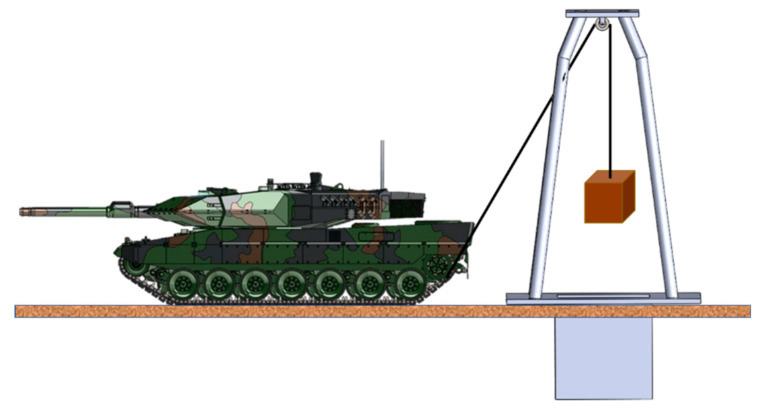
Schematic for the installation for measuring the moments of inertia of the transmission of a tracked vehicle, using the gravitational method.

The evaluation of the parameters necessary to determine the moments of inertia by the gravitational method Figure 2 is based on detaching the tracks from the drive wheel and attaching a cylindrical device. Its role is to wind a cable around it, which has a weight hanging from the other end.

The weight is hung by means of a pulley of a crane-type support. The working principle is as follows: the driver connects a stage of the hydromechanical transmission with an external pressure source, releases the brakes and the weight begins to fall. Knowing the weight value, the radius of the cylindrical device, the distance traveled by the weight and the fall time, analytical calculations can be performed to determine the moment of inertia [15]. Performing the same operations for both drive wheels will make it possible to determine the overall moment of inertia.

The experimental determination of the mass moment of inertia coefficient [16] aims to highlight its implications for the dynamic performance of the vehicle, in particular for the variation of speed, acceleration and space traveled during the starting process. Another parameter necessary to be verified is the dependence of the mass moment of inertia coefficient on the transmission ratio or the slips in the Hydroconverter.

The measured values of the moments of inertia of the parts that make up the main assemblies of the vehicle, which are in rotational motion, become input data in the general model for simulating the longitudinal dynamics. Based on these, the differential and algebraic equations of the virtual model approximate more precisely the actual operation process of the vehicle. There are a multitude of models for predicting longitudinal dynamics [17]. These models are found between the following extremes: the model regarding the mobility of vehicles on tracks [18] and the one that studies the interaction of the wheels (tracks) with the ground [19,20,21,22,23,24]. The final model (1) resulted from the processing of models for wheeled vehicles, as there is no unitary model for tracked military vehicles. Using the SIMSCAPE programming language, an own library was developed, which made the dynamic connection between armored housing, crawler propeller and ground. This model calculates, among other things, the forward resistances, which were not found in the Matlab toolbox. Based on these models, fast and robust algorithms were developed for evaluating acceleration performance and speeds under certain conditions. Tanks are complex structures, which create difficulties in establishing models. Among the algorithms developed and implemented in software, we mention: MOSES [21], NTVPM [22,23,24] and the least square method (LSM) [25].

The main new ideas of this paper can be summarized as follows: the building of a virtual model for the operation simulation of the accelerated motion regime of a hydromechanical transmission, monobloc, which equips a vehicle in operation; obtaining the analytical–numerical relation of the variation of the coefficient of inertia masses (*δ*) as a function of the pump speed; introduction of the correlation coefficient for an experimentally obtained quantity; development of two experimental methods for determining the coefficient of inertia masses; the concept developed and validated experimentally can be applied even in real operating conditions specific to military applications.

The paper is structured as follows: Section 2 addresses the hydrodynamic transmission model. Section 3 presents the algorithm for the analytical determination of the coefficient of inertia masses. Section 4 provides the model for simulating the variation of the coefficient of inertia masses. The experimental methods used to determine the same coefficient are presented in Section 5. Section 6 presents future developments. Finally, the conclusions of this paper are given in Section 7.

## 2. Background

To estimate the longitudinal dynamics of vehicles on tracks equipped with hydromechanical transmissions, a virtual model of operation for the accelerated motion regime was developed. The transmission is of hydromechanical type, monobloc.

The gears are engaged under load by actuating friction elements in the gearbox. Power is transmitted on one flow while driving in a straight line and on two power flows while turning. Rectilinear driving and turning are performed according to the graph representing the evolution for turn commands in Table 1.

**Figure 3:**
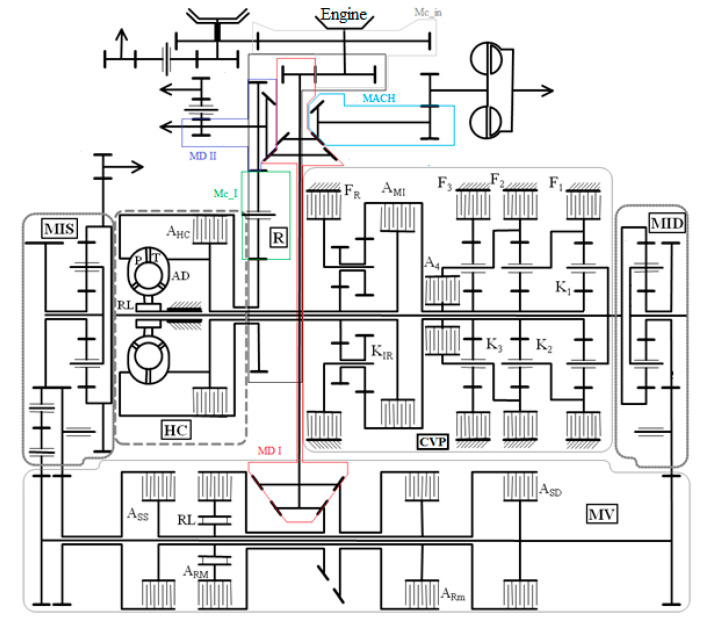
Kinematic diagram of the hydromechanical transmission [4].

According to the general kinematic diagram (Figure 3), the hydromechanical transmission consists of: input mechanism Mc_In, distribution mechanism I MD I, distribution mechanism II MD II, drive mechanism of the hydraulic coupling of the MACH fan, intermediate mechanism Mc_I, Hydroconverter HC, inverter mechanism MI, CVP planetary gearbox, MV turning mechanism and right and left MID and MIS planetary summation mechanisms.

## 3. Theoretical Considerations Regarding Mass Moment of Inertia Coefficient *δ*

The algorithm for determining the analytical expression that approximates the mass moment of inertia coefficient is based on mathematical models that describe the dynamic operation of the main subsystems of a vehicle. In order to carry out the calculations, it is necessary to elaborate the general nodal scheme of the vehicle, composed of individual nodal schemes (of the substructures) [25].

**Figure 4:**
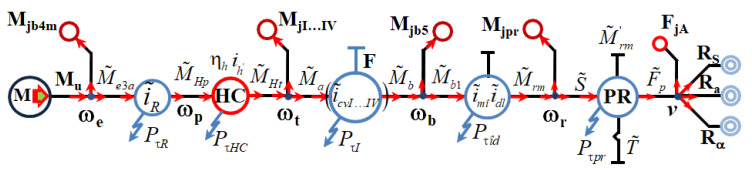
Generalized nodal scheme of a vehicle on tracks in the case of hydromechanical operation.

**Figure 5:**
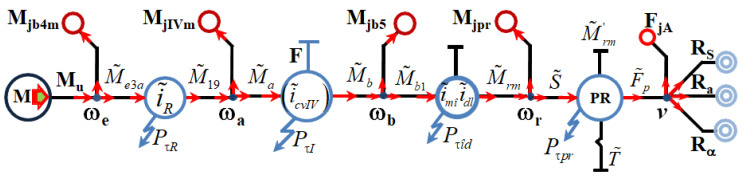
Generalized nodal scheme of a vehicle on tracks in the case of mechanical operation.

There are two variants of obtaining the analytical expression of the dynamic moment at the wheel: one in a hydrodynamic regime (Figure 4) and one in a mechanical regime (Figure 5).

The solution of operation in a hydrodynamic regime involves the application of the fundamental laws of the network model on the motor circuit–transmission–drive wheel.

The relation for determining the torque at the wheel in the case of a hydrodynamic regime is given by (1) [4]:

(1) $${\left({\tilde{M}}_{rm}\right)}_{i}={\left(M_{rm}\right)}_{i}-{\left(i_{cd}^{2}\right)}_{i}\cdot {\left(\eta_{cd}\right)}_{i}\cdot \left[{\left(I_{TR}\right)}_{i}+I_{EP}\cdot K_{h}\cdot \left(\frac{d\omega_{p}}{d\omega_{t}}\right)\right]\cdot \frac{d\omega_{r}}{dt}\;\left[\mathrm{Nm}\right]$$

where: ${\left(M_{rm}\right)}_{i}={\left(i_{cv}\right)}_{i}\cdot i_{mî}\cdot i_{dl}\cdot {\left(\eta_{cv}\right)}_{i}\cdot \eta_{mî}\cdot \eta_{dl}\cdot M_{Ht}\;\left[\mathrm{Nm}\right]$, ${\left(i_{cd}\right)}_{i}={\left(i_{cv}\right)}_{i}\cdot i_{mî}\cdot i_{dl}\;\left[-\right]$, ${\left(\eta_{cd}\right)}_{i}={\left(\eta_{cv}\right)}_{i}\cdot \eta_{mî}\cdot \eta_{dl}\;\left[-\right]$, $I_{EP}=I_{b4}\cdot i_{R}^{2}\cdot \eta_{R}\;\left[\mathrm{kg}\cdot m^{2}\right]$, ${\left(I_{TR}\right)}_{i}=\left[I_{i}+I_{mi}+\frac{I_{b5}}{{\left(i_{cv}^{2}\right)}_{i}\cdot {\left(\eta_{cv}\right)}_{i}}\right]\;\left[\mathrm{kg}\cdot m^{2}\right]$.

The second solution for operation in a mechanical regime involves following a different route composed of ground–tracks–drive wheel.

The relation for determining the momentum at the drive wheel in the case of a mechanical regime is given by (2):

(2) $${\left({\tilde{M}}_{rm}\right)}_{IVm}={\left(M_{rm}\right)}_{IVm}-{\left(i_{t}^{2}\right)}_{IV}\cdot {\left(\eta_{t}\right)}_{IV}\cdot {\left(I_{t}\right)}_{IVm}\cdot \frac{d\omega_{r}}{dt}\;\left[\mathrm{Nm}\right]$$

(3) $${\tilde{M}}_{rm}={\tilde{F}}_{p}\cdot r_{rm}+I_{pr}\cdot \frac{d\omega_{r}}{dt}+R_{pr}\cdot r_{rm}\;\left[\mathrm{Nm}\right]$$

where: ${\left(i_{t}\right)}_{IV}=i_{R}\cdot {\left(i_{cv}\right)}_{IV}\cdot i_{mî}\cdot i_{dl}\;\left[-\right]$; ${\left(\eta_{t}\right)}_{IV}=\eta_{R}\cdot {\left(\eta_{cv}\right)}_{IV}\cdot \eta_{mî}\cdot \eta_{dl}\;\left[-\right]$.

For the operation of the vehicle in a mechanical regime, the expression for determining the torque in dynamic mode is given by (3).

From Equations (1)–(3), the following relations can be deduced for the dynamic traction force:

(4) $${\tilde{F}}_{t}={\tilde{F}}_{p}+\frac{I_{pr}}{r_{rm}^{2}}\cdot \frac{dv}{dt}+R_{pr}=R_{s}+R_{\alpha }+R_{a}+F_{jA}\;\left[N\right]$$

where $F_{jA}\;\left[N\right]$ is the load factor of the inertial flow of the vehicle,

(5) $$F_{jA}=\left(M+m_{ş}\right)\cdot \frac{dv}{dt}\;\left[N\right]$$

and for the two modes of operation we will have:

- hydromechanical operation:
  (6) $${\left({\tilde{F}}_{t}\right)}_{i}={\left(F_{t}\right)}_{i}-\frac{{\left(i_{cd}^{2}\right)}_{i}\cdot {\left(\eta_{cd}\right)}_{i}}{r_{rm}^{2}}\cdot \left[{\left(I_{TR}\right)}_{i}+I_{EP}\cdot K_{h}\cdot \frac{d\omega_{p}}{d\omega_{t}}\right]\cdot \frac{dv}{dt}\;\left[N\right]$$
- mechanical operation—HC blocked:
  (7) $${\left({\tilde{F}}_{t}\right)}_{IVm}={\left(F_{t}\right)}_{IVm}-\frac{{\left(i_{cd}^{2}\right)}_{IV}\cdot {\left(\eta_{cd}\right)}_{IV}}{r_{rm}^{2}}\cdot {\left(I_{t}\right)}_{IVm}\cdot \frac{dv}{dt}\;\left[N\right]$$

Since the power flows from the engine and the one from the track must be equal, this results in (4) = (6) = (7), which is the equation of dependence between the traction force specific to the steady state of operation and the dynamic propulsion force. By replacing the dynamic propulsion force in the dependency equation, the analytical expression of the traction force is specific to the stationary mode of operation and the results. On the other hand, the traction force specific to the stationary operating regime is also defined by the equation of the traction balance:

(8) $${\left(F_{t}\right)}_{i}=R_{pr}+R_{s}+R_{\alpha }+R_{a}+{\left(\delta \right)}_{i}\cdot M_{a}\cdot \frac{dv}{dt}\;\left[N\right]$$

**Figure 6:**
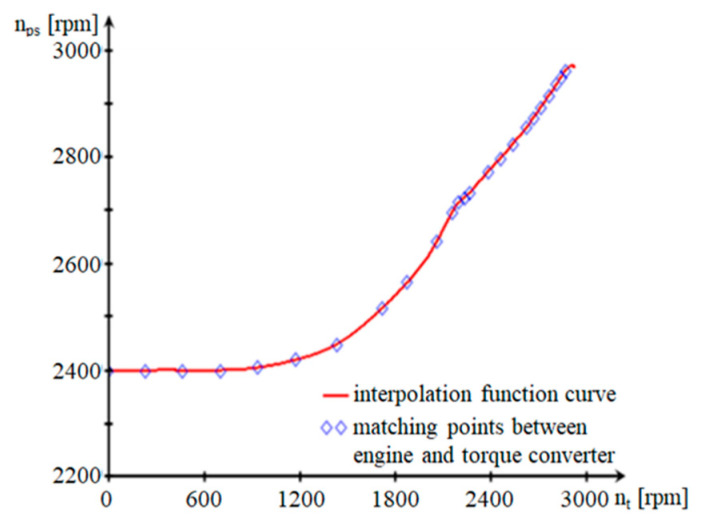
Dependence between pump speed and turbine speed.

Identifying the terms of the two defining relations of the traction force in the stationary operating regime (Figure 6), the analytical expression of (*δ*) for the mechanical and hydromechanical operation is determined:

(9) $${\left(\delta \right)}_{IVm}=\left[1+\frac{m_{ş}}{M_{a}}+\frac{I_{pr}}{M_{a}\cdot r_{rm}^{2}}+\frac{{\left(i_{t}^{2}\right)}_{IV}\cdot {\left(\eta_{t}\right)}_{IV}}{M_{a}\cdot r_{rm}^{2}}\cdot {\left(I_{t}\right)}_{IVm}\right]\;\left[-\right]$$

(10) $${\left(\delta \right)}_{i}=1+\frac{m_{ş}}{M_{a}}+\frac{I_{pr}}{M_{a}\cdot r_{rm}^{2}}+\frac{{\left(i_{cd}^{2}\right)}_{i}\cdot {\left(\eta_{cd}\right)}_{i}}{M_{a}\cdot r_{rm}^{2}}\cdot \left[{\left(I_{TR}\right)}_{i}+I_{EP}\cdot K_{h}\cdot \frac{d\omega_{p}}{d\omega_{t}}\right]\;\left[-\right]$$

**Figure 7:**
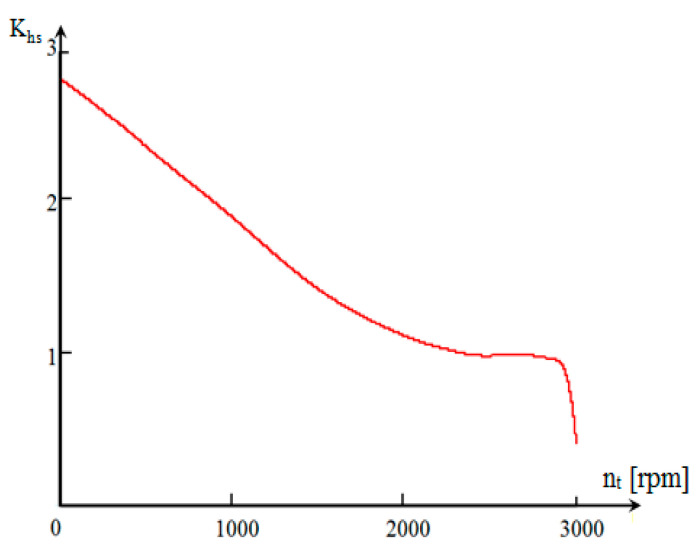
The dependence between the transformation ratio and turbine speed.

**Figure 8:**
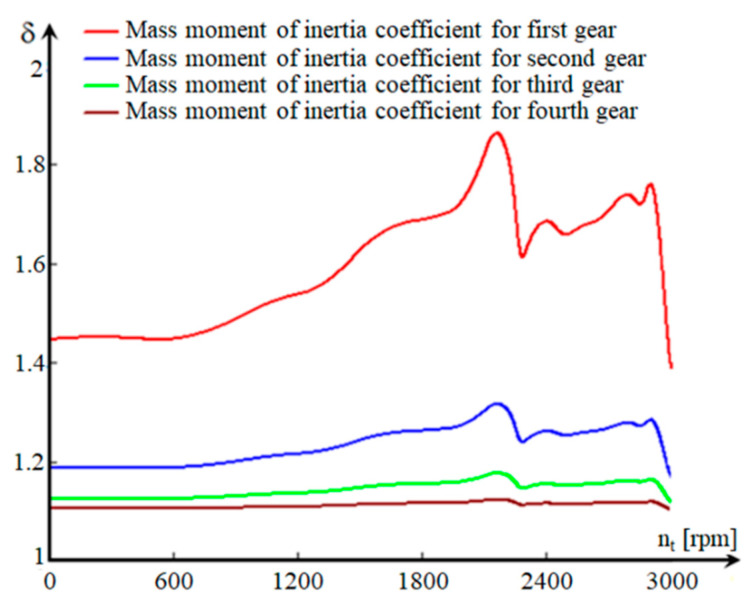
Variation of the mass moment of inertia coefficient (*δ*) depending on the turbine speed.

Following the replacement of the expressions for the angular velocities of the pump (Figure 7) and the turbine (Figure 8) in (9) and (10), the final relation of *δ* for hydromechanical operation is:

(11) $${\left(\delta \right)}_{i}=1+\frac{m_{ş}}{M_{a}}+\frac{I_{pr}}{M_{a}\cdot r_{rm}^{2}}+\frac{{\left(i_{cd}^{2}\right)}_{i}\cdot {\left(\eta_{cd}\right)}_{i}}{M_{a}\cdot r_{rm}^{2}}\cdot \left[{\left(I_{TR}\right)}_{i}+I_{EP}\cdot K_{h}\cdot \frac{dn_{p}}{dn_{t}}\right]\;\left[-\right]$$

(12) $$\{\begin{array}{l} n_{ps}\left(n_{t}\right)=\mathrm{interp}\left[cspline\left(n_{t},n_{c}\right),n_{t},n_{c}\right]\\ {\left[\delta \left(n_{t}\right)\right]}_{i}=1+\frac{m_{ş}}{M_{a}}+\frac{I_{pr}}{M_{a}\cdot r_{rm}^{2}}+\frac{{\left(i_{cd}^{2}\right)}_{i}\cdot {\left(\eta_{cd}\right)}_{i}}{M_{a}\cdot r_{rm}^{2}}\cdot \left[{\left(I_{TR}\right)}_{i}+I_{EP}\cdot K_{hs}\left(n_{t}\right)\cdot \frac{dn_{ps}\left(n_{t}\right)}{dn_{t}}\right]\;\left[-\right] \end{array}$$

The variation (*δ*) is not important for speeds between 0 and 700 rpm, regardless of the floor on which the vehicle operates (Figure 7). During this interval, there are large slips in the hydroconverter, which corresponds to the specific situation of starting the vehicle. For speeds in the range of 700–2160 rpm, (*δ*) increases. This range corresponds to the area in which the aggregate operates as a hydroconverter. An area in which the value (*δ*) is decreasing appears during the transition from hydroconverter to clutch mode. This is due to the decrease in the slip rate of the pump in relation to the turbine (expressed by the derivative of the pump speed in relation to the turbine speed), as a result of the transition of the hydro unit from the hydroconverter to clutch mode. Since we have an MBS, we consider, for the study of dynamism performances, that the algorithms for identifying the parameters, namely the LSM, are easy to implement. This method allows us to update the current estimates of the tracked parameters [25].

The “atypical” variation of (*δ*) is also due to the mode of transition from one regime to another of the transformation ratio function. The turbine speed is directly proportional to the speed of the vehicle, therefore, the dependence (*δ*) can also be expressed as a function of the speed of the vehicle, by changing the variable $n_{t}\to v$, so that the speed of the vehicle can be determined according to turbine speed:

(13) (v)i=π·nt·rrm30·(icv)i·imî·idl→(v)i=π·nt·rrm30·(icd)i [ms]

**Figure 9:**
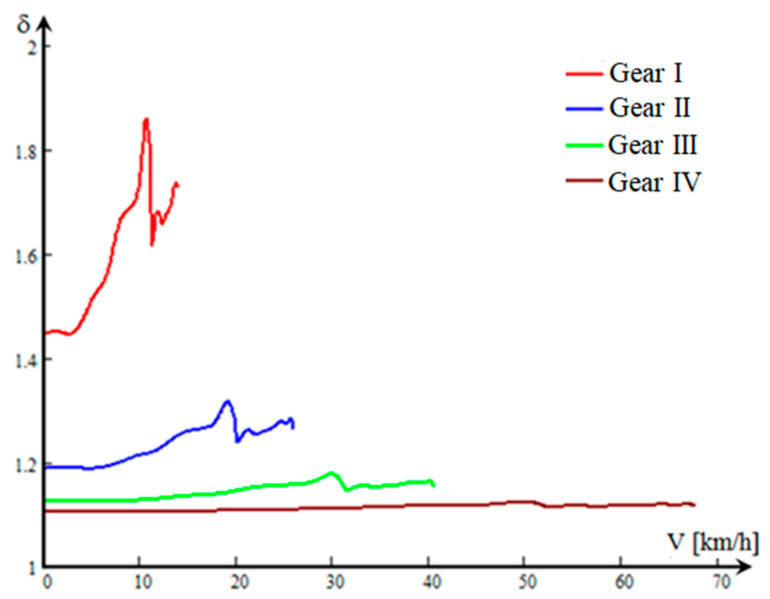
Variation of the mass moment of inertia coefficient depending on the speed of the vehicle.

Therefore, the expression of (*δ*) can be defined as a function of *v* (Figure 9) for the four stages of the CVP:

(14) $${\left[\delta \left(v\right)\right]}_{i}=1+\frac{m_{ş}}{M_{a}}+\frac{I_{pr}}{M_{a}\cdot r_{rm}^{2}}+\frac{{\left(i_{cd}^{2}\right)}_{i}\cdot {\left(\eta_{cd}\right)}_{i}}{M_{a}\cdot r_{rm}^{2}}\cdot \left[{\left(I_{TR}\right)}_{i}+I_{EP}\cdot K_{hs}\left(v\right)\cdot \frac{1}{\frac{30\cdot {\left(i_{cd}\right)}_{i}}{3,6\cdot \pi \cdot r_{rm}}}\cdot \frac{dn_{ps}\left(v\right)}{dv}\right]\;\left[-\right]$$

The higher values of (*δ*) in the lower stages are due to the transmission ratio of the mechanical transmission and the equivalent reduced moment of inertia to the wheel axle $\left(I_{TR}\right)$.

## 4. Concepts for Simulating the Variation of the Mass Moment of Inertia Coefficient *δ*

In order to indirectly determine the mode of variation of (*δ*), some of the data obtained from simulation process of the longitudinal dynamics of a vehicle are needed, namely the signals specific to the terms that are found in the differential equation of motion:

(15) a(t)=dvdt=gδ(t)·F(t)t−Rprsaα(t)Ga [ms2]⇒δ(t)=F(t)t−Rprsaα(t)a(t)·Ma [−]

**Figure 10:**
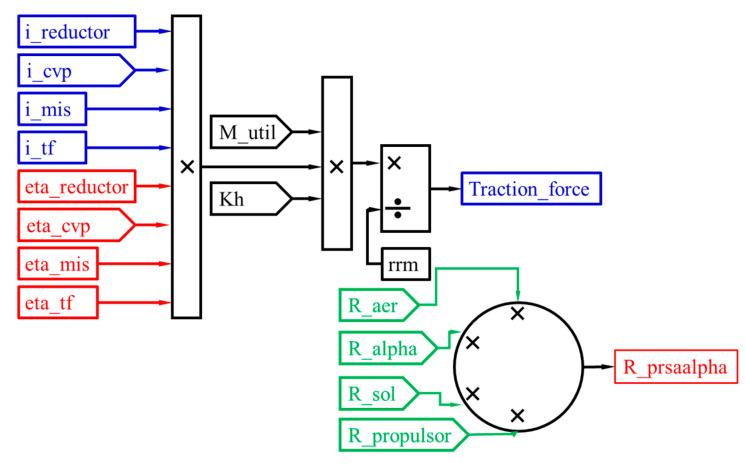
Modeling of traction force and forward resistance forces.

The traction force signal modeling is performed by utilizing, with the SIMULINK programming language, the ratio between the torque at the drive wheel, determined as a result of the product between the signals specific to the torque at the engine M_util, the transformation ratio of the hydroconverter $K_{h}$, transmission ratios and element yields arranged between the engine and the drive wheel and the signal input for modeling the radius of the drive wheel $r_{rm}$. The forward resistance force signal is obtained by summing the thrust resistance force R_propulsor, the rolling resistance force R_sol, the air resistance force R_aer and the climbing resistance force R_alpha (Figure 10).

**Figure 11:**
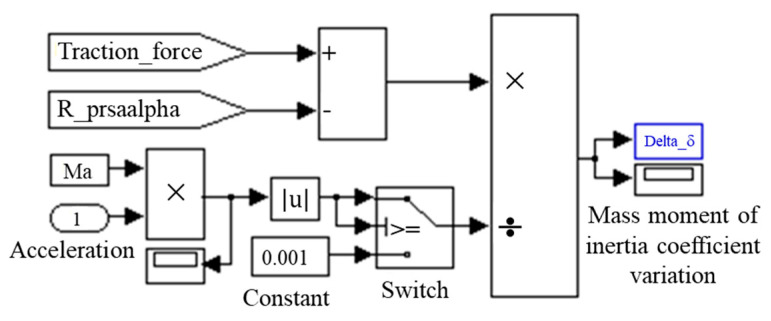
Modeling the mass moment of inertia coefficient.

Longitudinal acceleration signal *acceleration*, that of the mass of the vehicle *Ma*, as well as the signals introduced by the blocks that model the traction force and the one of forward resistance, are used to model the expression of the mass moment of inertia coefficient (Figure 11).

## 5. Experimental Methods Used to Determine the Mass Moment of Inertia Coefficient *δ*

**Figure 12:**
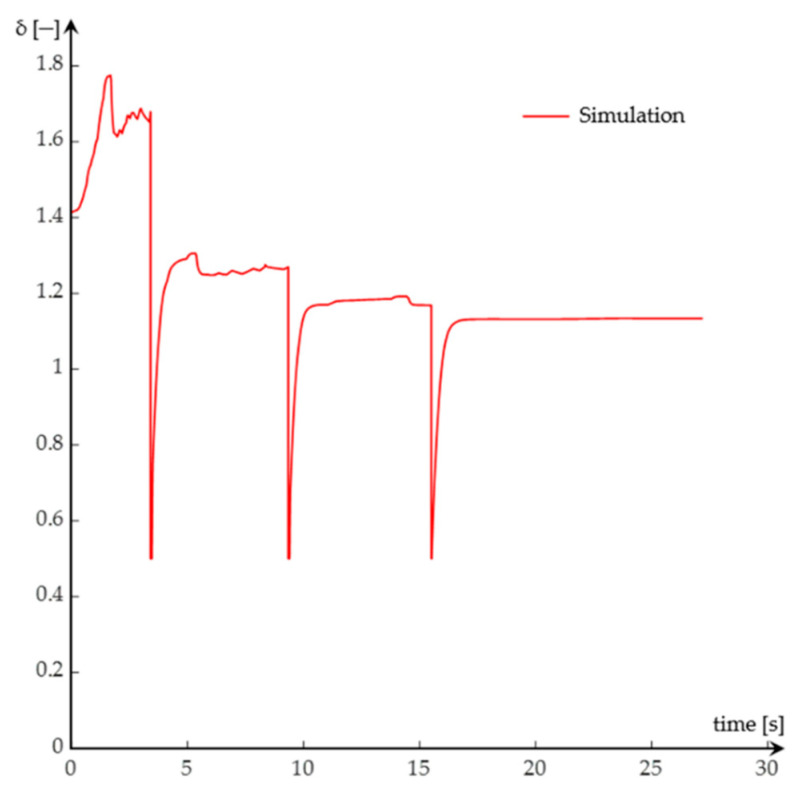
The variation graph of (*δ*) during the simulation of the starting process.

Experimental methods highlight the mode of variation of (*δ*) by determining the values of equivalent moments of inertia entering Expression (16) (Figure 12).

For the experimental determination of the equivalent reduced moments of inertia values *I_pr_*, *I_EP_*, *I_TR_* and *I_t_*, two methods were used: the gravitational one and the three-wire suspension.

(16) $$\{\begin{array}{l} \delta_{i}=1+\frac{m_{ş}}{M_{a}}+\frac{I_{pr}}{M_{a}\cdot r_{rm}^{2}}+\frac{{\left(i_{cd}^{2}\right)}_{i}\cdot {\left(\eta_{cd}\right)}_{i}}{M_{a}\cdot r_{rm}^{2}}\cdot \left[{\left(I_{TR}\right)}_{i}+I_{EP}\cdot K_{h}\cdot \frac{d\omega_{p}}{d\omega_{t}}\right]\;\left[-\right]\\ \delta_{IVm}=1+\frac{m_{ş}}{M_{a}}+\frac{I_{pr}}{M_{a}\cdot r_{rm}^{2}}+\frac{{\left(i_{t}^{2}\right)}_{IV}\cdot {\left(\eta_{T}\right)}_{IV}}{M_{a}\cdot r_{rm}^{2}}\cdot {\left(I_{t}\right)}_{IVM}\;\left[-\right] \end{array}$$

### 5.1. The Gravitational Method

The gravitational method is a means of determining the equivalent reduced moment of inertia at the drive wheel of all rotating parts, arranged between the Hydroconverter turbine and the drive wheel.

**Figure 13:**
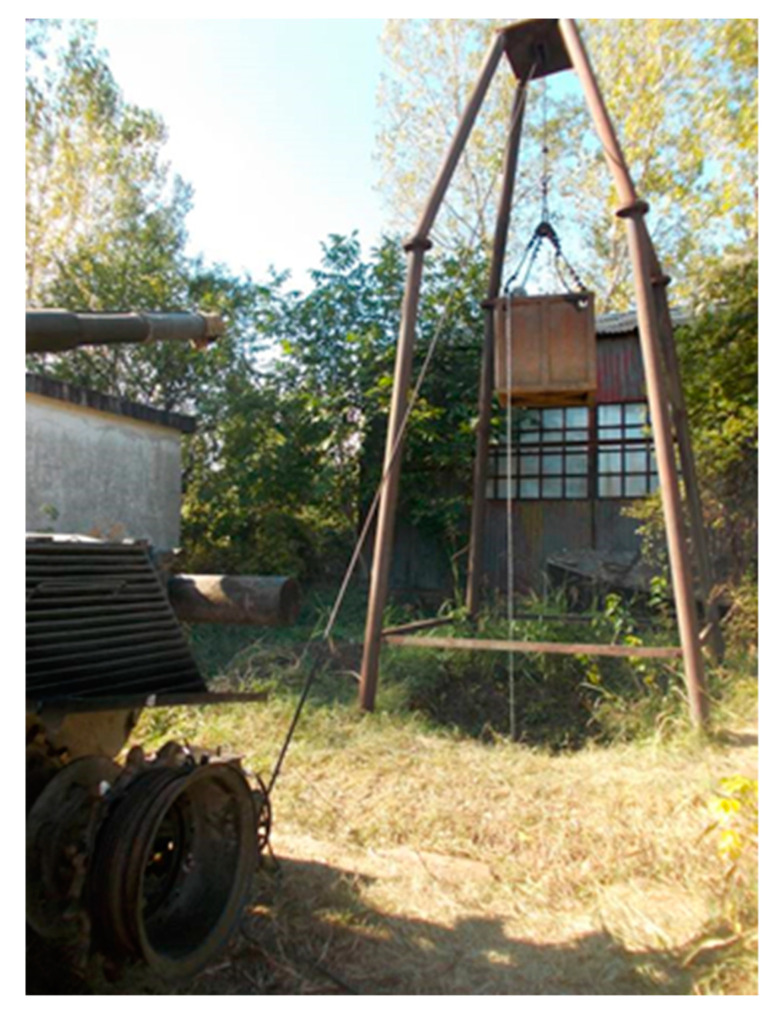
The experimental device used for determination of (*δ*).

The equipment and materials used consist of an RS 38 incremental encoder, an encoder mounting device, a Monarch-type ATC frequency meter, a DMC 9012A measuring bridge, a mobile stand to check the hydraulic installation, a Power Book 1400cs portable electronic computer, connection cables, extension cords and power cables from the 220 V mains, a drum, a stopwatch and an experimental assembly (Figure 13).

The experimental assembly consists of a metal frame, a pulley, and a metal box in which marked weights are inserted. The cable is attached at one end to the metal box and at the other end by a drum fixed to the gear of the drive wheel.

The drum is designed to avoid uneven winding of the cable and the friction of the cable by the structural elements of the vehicle.

**Figure 14:**
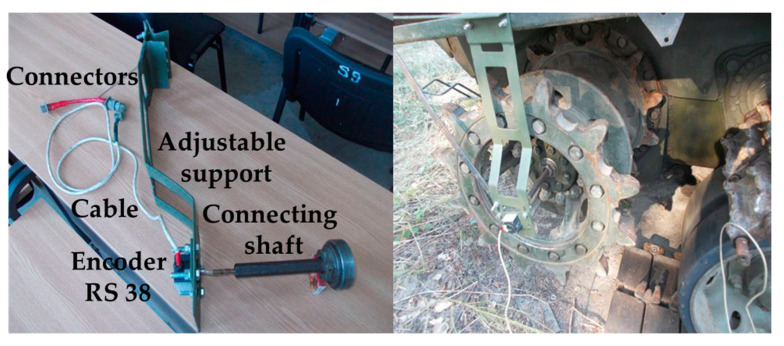
Mounting the RS-38 optical encoder on the right-hand drive wheel.

To measure the speed of the drive wheel, an encoder is mounted on an adjustable support on the drive wheel on the other side of the vehicle (Figure 14).

**Figure 15:**
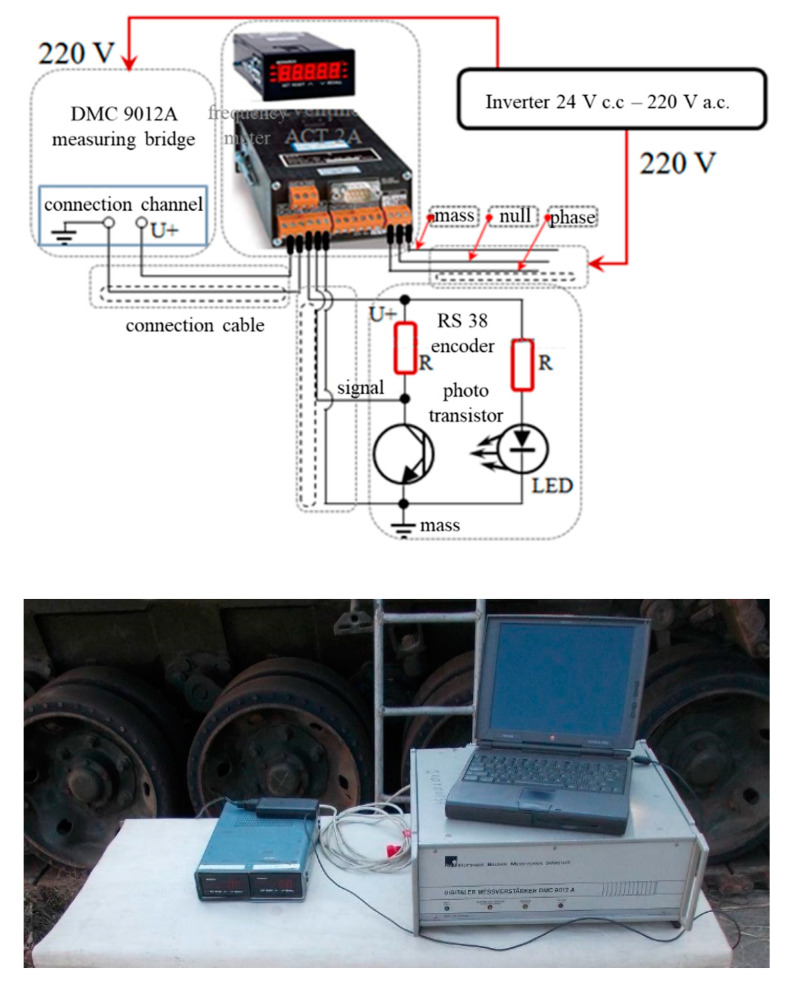
Measuring chain (*δ*): DMC 9012A bridge, frequency meter, laptop.

The driving of the RS38 optical encoder is performed by means of a connecting shaft, provided with an elastic coupling, compensating for coaxial deviations, connected to the shaft of the final transmission. In Figure 15 we represent the measuring chain.

#### 5.1.1. The Classical Gravitational Method

**Figure 16:**
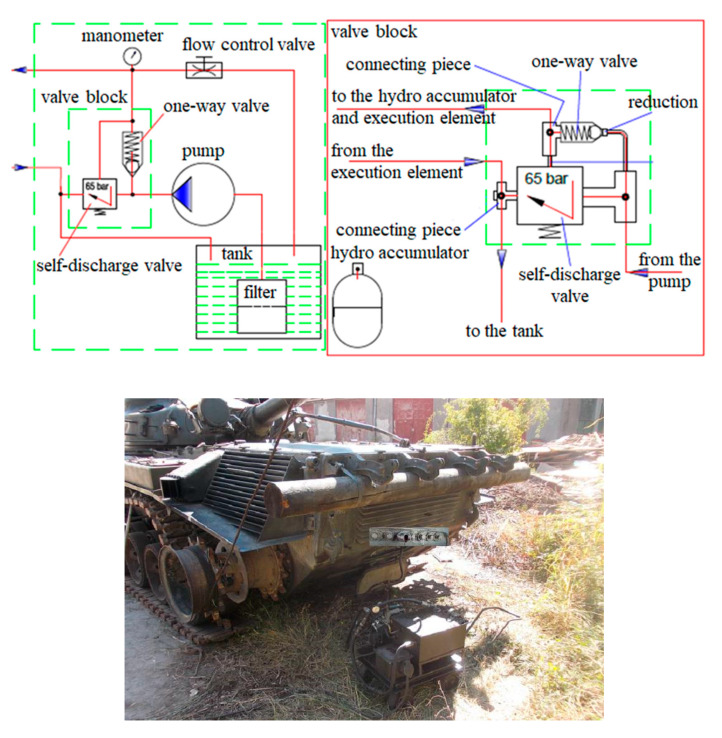
External gearbox coupling system.

The tests are performed under the following conditions: the engine is stopped and disconnected from the transmission. As stopping the engine results in a lack of pressure in the hydraulic control system, an external pressure source is used to connect the transmission gears (Figure 16).

**Figure 17:**
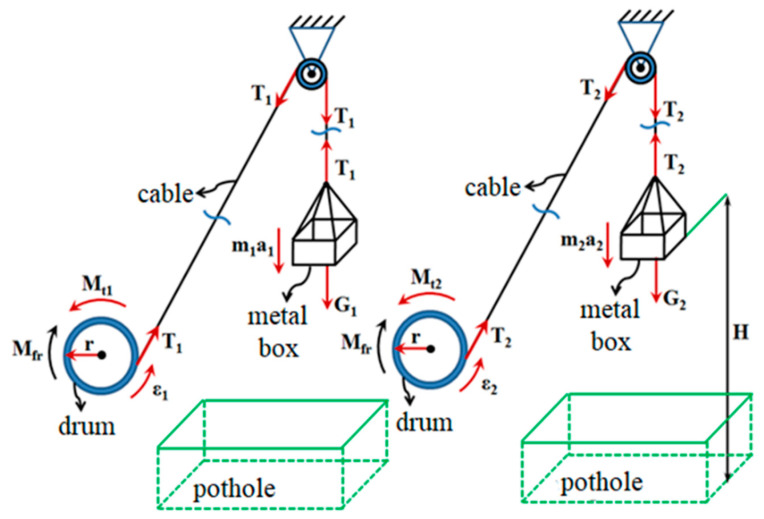
The classical gravitational method, the representation of forces and moments.

The test consists in the descent of the metal box, in a uniformly accelerated regime, with a known acceleration *a*, until it crosses the height *H* and reaches the ground. The rotational motion of the drive wheel and the rest of the transmission components has the character of a uniformly accelerated motion, characterized by the angular acceleration *ε* (Figure 17).

Thus, we can define the equations that describe the two uniformly accelerated movements, of rotation and translation, for two different loads, *m*_1_ and *m*_2_:

(17) $$\{\begin{array}{ll} m_{1}\cdot g-T_{1}=m_{1}\cdot a_{1} & \;m_{2}\cdot g-T_{2}=m_{2}\cdot a_{2}\\ I_{echiv\_rm}\cdot \varepsilon_{1}=M_{t1}-M_{fr} & \;I_{echiv\_rm}\cdot \varepsilon_{2}=M_{t2}-M_{fr}\\ M_{t1}=T_{1}\cdot r & \;M_{t2}=T_{2}\cdot r \end{array}\;\left[\mathrm{Nm}\right]$$

The following assumptions are made: the losses generated by friction are identical regardless of the value of the weight of the metal box and the movement of the box, the drum is uniformly accelerated, and the initial speed is zero. The hypotheses show:

(18) {a1=2Ht12 [ms2] a2=2Ht22 [ms2]ε1=1r·2Ht12 [1s2] ε2=1r·2Ht22 [1s2]

From Relations (17) and (18), the expression of the equivalent moment of inertia is found, reduced to the drive wheel:

(19) $$I_{echiv\_classical\;method}=\frac{r^{2}\cdot t_{1}^{2}\cdot t_{2}^{2}\cdot \left(G_{2}-G_{1}\right)}{2\cdot H\cdot \left(t_{1}^{2}-t_{2}^{2}\right)}-\frac{r^{2}\cdot \left(G_{2}\cdot t_{1}^{2}-G_{1}\cdot t_{2}^{2}\right)}{g\cdot \left(t_{1}^{2}-t_{2}^{2}\right)}\;\left[\mathrm{kg}\cdot m^{2}\right]$$

The experimental data (Table 2) are entered in Relation (19) and the values corresponding to the equivalent moments of reduced inertia at the drive wheel are obtained (Table 3).

#### 5.1.2. Computer-Assisted Gravitational Method

**Figure 18:**
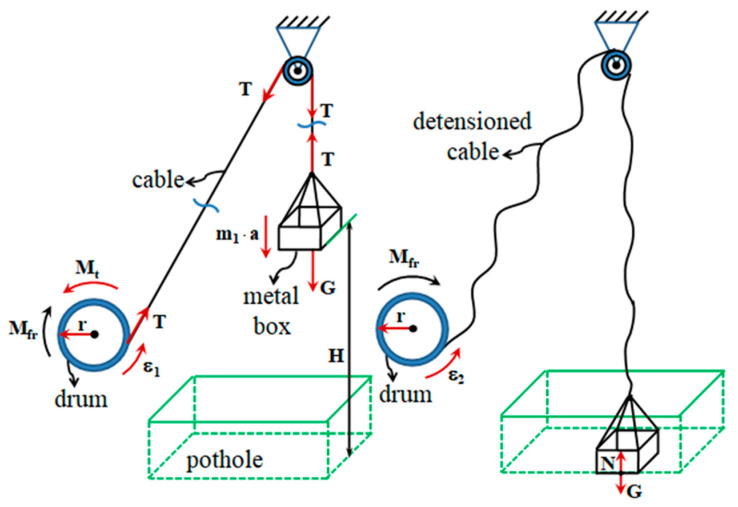
Computer-assisted gravitational method, representation of forces and moments.

The experimental determination of the equivalent reduced moment of inertia at the drive wheel *I_TR_* using the computer-assisted gravitational method is like the classical method. The stages of the procedure for determining the moments of inertia and the resulting equations are different. In this situation, only one weight of mass *m*_1_ is used, which falls freely for the distance *H*, generating a uniformly accelerated motion, characterized by the acceleration *a*. Additionally, due to the stretching phenomenon, tension appears in the metal cable that acts on the drum with the moment *M_t_* and imprints the angular acceleration *ε*_1_ on the rotating parts. The angular acceleration *ε*_1_ and the linear acceleration *a* are quantities valid only during the acceleration phenomenon, from the moment the box is released until the ground is touched by the lower part of the box (Figure 18).

From the moment the box touches the ground, the deceleration phenomenon begins, the tension in the metal cable has zero value and the drum and the rotating elements are characterized by the deceleration *ε*_2_.

(20) {m1·g−T1=m1·a [Nm]Iechiv_rm·ε1=Mt−Mfr [Nm]Mt=T1·r [Nm]a=ε·r [ms2]

The rotational movement of the drum, including the elements arranged between the drum and the turbine of the Hydroconverter, due to the constant friction moment *M_fr_*, is gradually slowed down, finally reaching rest. In this situation, the equation describing the deceleration phenomenon of the drum and the rotating parts is:

(21) {Iechiv_rm·ε2=−Mfr [Nm]ε=dωdt [1s2]

Equations (20) and (21) result in:

(22) $$I_{echiv\_rm\_as\_computer}=\frac{m_{1}\cdot r\cdot \left(g-\varepsilon_{1}\cdot r\right)}{\varepsilon_{1}-\varepsilon_{2}}\;\left[\mathrm{kg}\cdot m^{2}\right]$$

**Figure 19:**
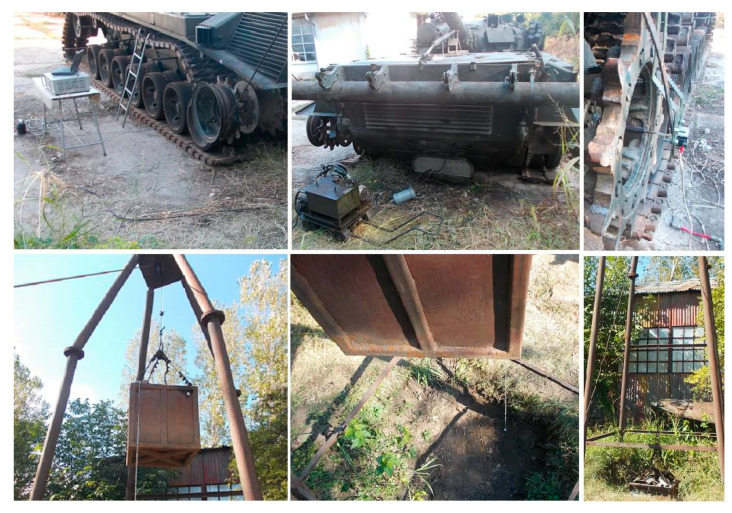
Testing using the computer-assisted gravitational method.

The tests are performed successively for operation in the neutral position and in the four gears of the planetary gearbox. The data acquisition system measures the values of the angular velocity of the drive wheel until it stops. The measurements are repeated three times for each stage of the planetary gearbox (Figure 19). This method does not require measurements with two different masses. It is enough that the measurements are performed with only one mass, *m*_2_. To confirm the quality of the data obtained during the previous tests, in the case of I and II gears, the mass *m*_1_ is used.

**Figure 20:**
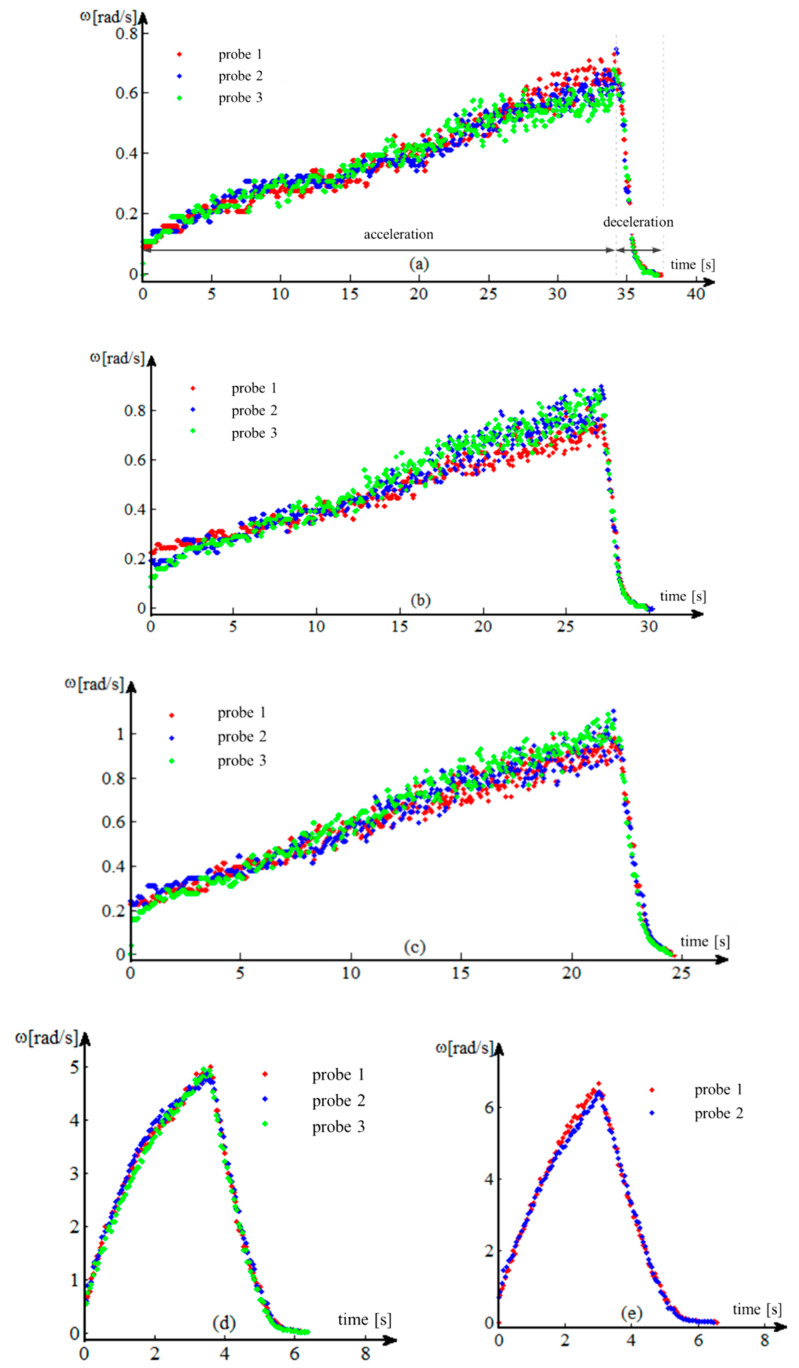
Variation of the angular speed of the drive wheel. (**a**) Operation in I gear; (**b**) operation in II gear; (**c**) operation in III gear; (**d**) operation in IV gear; (**e**) operation in neutral.

The processed data [26,27] allow for the graphical tracing of the variation of the angular velocity, during the acceleration and deceleration of the transmission parts, in the form of variation of the angular velocity of the drive wheel (Figure 20a–e).

A first analysis of the previous graphs shows an almost linear evolution of the angular velocity *ω*. The phenomenon is more evident in the acceleration phases of the first three gears and in all the deceleration phases of all gears (except their terminal part).

The nonlinearity noticed in the final acceleration phase of the 4th gear and in the terminal phases of the decelerations of all gears (exponentially) is due to the frictions in the system, both the turbulent one in the Hydroconverter and the laminar one in the rest of the transmission. One of the hypotheses imposed is that friction has a linear character in relation to speed, so the frictions due to the turbulent flow phenomena in the Hydroconverter are linearized. This allowed for a linear interpretation of the angular velocity behavior.

**Figure 21:**
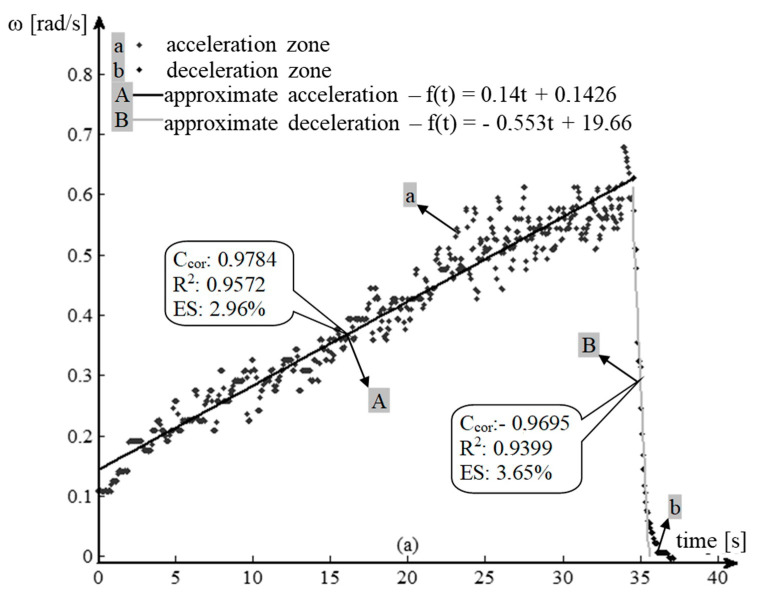

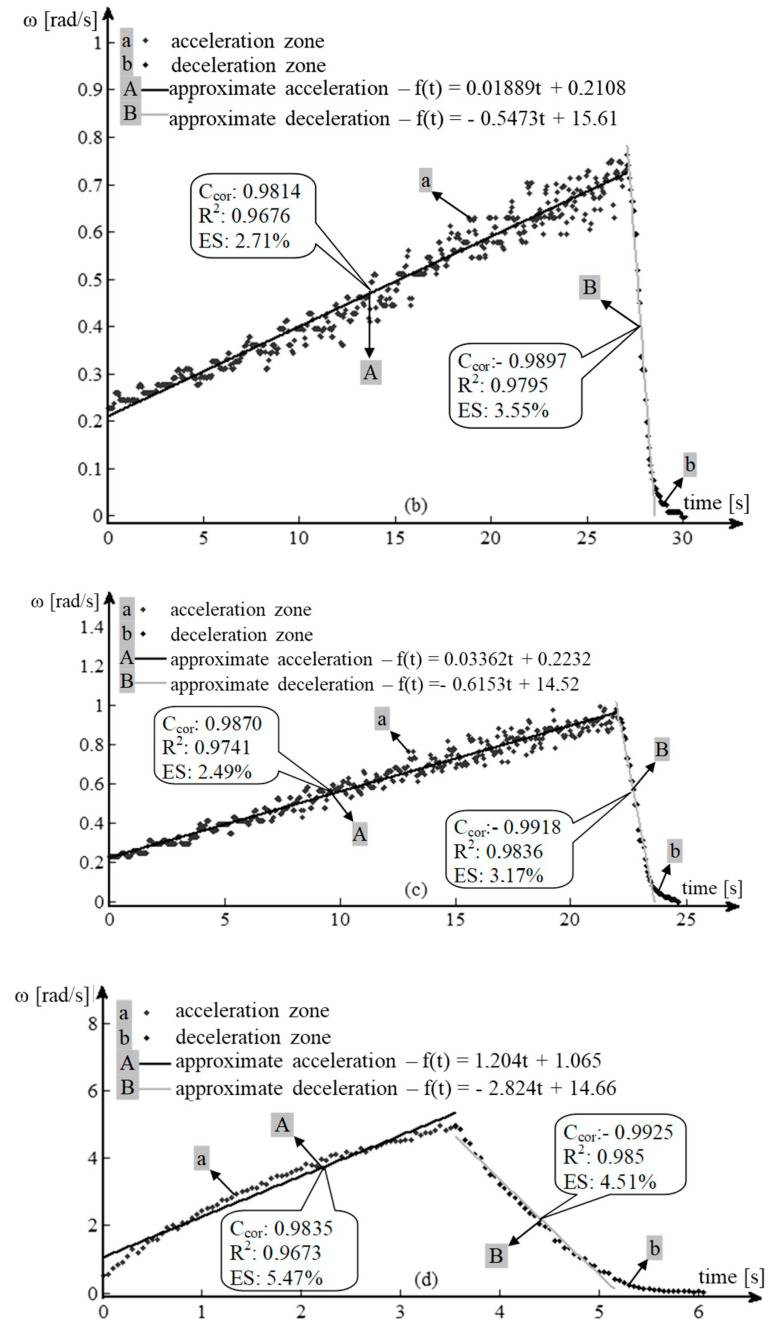

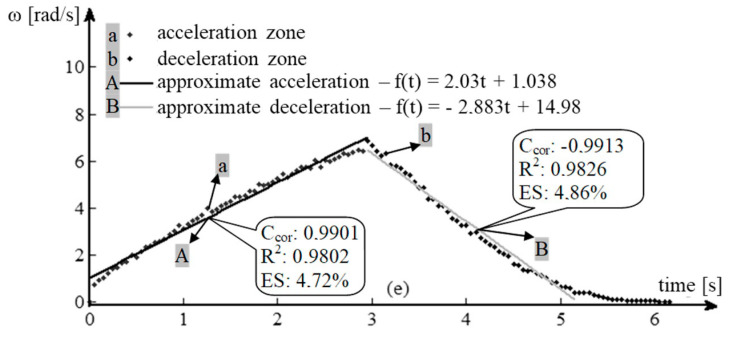
Experimental data processing. Modeling accuracy parameters. (**a**) I gear, probe 3; (**b**) II gear, probe 1; (**c**) III gear, probe 1; (**d**) IV gear, probe 3; (**e**) neutral, probe 1.

The linearity of the behavior can be highlighted by several methods. One of these is the use of the correlation coefficient. In general, the correlation coefficient is used to determine how a signal measured at one point in a system tracks the evolution of another signal measured at either the same point in the system or at another. In this situation, the closer the correlation coefficient is to one, the better the signals are correlated. The application of this method in this case aims to establish the linearity of the evolution of the angular velocity, by using the “vector” time, whose evolution is independent, “correlated” with the evolution of the angular velocity vector. A correlation coefficient as close as possible to the unit value leads to the conclusion that the dependent variable (angular velocity) also has a linear evolution. To approximate the angular velocity for the two operating modes (acceleration, deceleration), the method of least squares is used (Figure 21a–e).

(23) $$C_{cor}=\frac{C_{\mathrm{cov}}\left(t,\omega \right)}{S_{t}\cdot S_{\omega }}=\frac{\frac{\sum_{i=1}^{n}\left(t_{i}-\bar{t}\right)\cdot \left(\omega_{i}-\bar{\omega }\right)}{n-1}}{\sqrt{\frac{\sum_{i=1}^{n}{\left(t_{i}-\bar{t}\right)}^{2}}{n-1}}\cdot \sqrt{\frac{\sum_{i=1}^{n}{\left(\omega_{i}-\bar{\omega }\right)}^{2}}{n-1}}}\;\left[-\right]\;\in \;\left[-1,1\right]$$

The value of the coefficient of determination (24) is close to the unit value for all samples, which means that a high percentage of the experimental data are very close to the calculated values, as follows:

- acceleration phase—95.72% in I gear, 96.76% in II gear, 97.41% in III gear, 96.73% in IV gear and 98.02% in neutral;
- deceleration phase—93.99% in I gear, 97.95% in II gear, 98.36% in III gear, 98.50% in IV gear and 98.26% in neutral.

(24) $$R^{2}=1-\frac{\sum_{i=1}^{n}\left(\omega_{i}-{\hat{\omega }}_{i}\right)}{\sum_{i=1}^{n}\left(\omega_{i}-\bar{\omega }\right)}\;\left[-\right]$$

Another relevant criterion for the quality of the approximation of the experimental data is the standard error ES (25) which falls within the allowed approximation limits:

- I gear—2.96% acceleration phase and 3.65% deceleration phase;
- II gear—2.71% acceleration phase and 3.55% deceleration phase;
- III gear—2.49% acceleration phase and 3.17% deceleration phase;
- IV gear—5.47% acceleration phase and 4.51% deceleration phase;
- neutral—4.72% acceleration phase and 4.86% deceleration phase.

(25) $$R^{2}=1-\frac{\sum_{i=1}^{n}\left(\omega_{i}-{\hat{\omega }}_{i}\right)}{\sum_{i=1}^{n}\left(\omega_{i}-\bar{\omega }\right)}\;\left[-\right]$$

Based on the algorithm for processing and representing the angular velocities as well as the angular accelerations and decelerations [1,28,29,30], the corresponding values are calculated for each stage of the gearbox and for each test separately (Table 4).

Due to the behavioral nonlinearities of the angular velocity, the last section of the evidence is not taken into account when obtaining the angular decelerations.

The linear approximation of the entire deceleration area to the actual stop of the drive wheel, including non-linear areas, could introduce errors in the calculation of the angular deceleration.

For this reason, the model is used only to describe the linear area of the data section, where the angular velocity decreases rapidly after an almost linear variation (Table 5).

### 5.2. Three-Wire Suspension Method

This method allows for the experimental determination of the moments of inertia for various rotating moving parts.

(26) $$I_{pr}=\sum_{i=1}^{2}I_{rî}\cdot {\left(\frac{r_{rm}}{r_{rî}}\right)}^{2}+\sum_{i=1}^{12}I_{g}\cdot {\left(\frac{r_{rm}}{r_{g}}\right)}^{2}+\sum_{i=1}^{6}I_{rs}\cdot {\left(\frac{r_{rm}}{r_{rs}}\right)}^{2}\;\left[\mathrm{kg}\cdot m^{2}\right]$$

(27) $$I_{EP}=\left(\begin{array}{l} I_{e}i_{R}^{2}\eta_{R}+\frac{i_{R}^{2}\eta_{R}}{i_{1}^{2}\eta_{1}}I_{5}+\frac{i_{R}^{2}\eta_{R}}{i_{2}^{2}\eta_{2}}I_{6}+\frac{i_{R}^{2}\eta_{R}}{i_{2}^{2}\eta_{2}i_{3}^{2}\eta_{3}}I_{7}+\frac{i_{R}^{2}\eta_{R}}{i_{2}^{2}\eta_{2}i_{5}^{2}\eta_{5}i_{7}^{2}\eta_{7}}I_{8}+\\ +\frac{i_{R}^{2}\eta_{R}}{i_{2}^{2}\eta_{2}i_{10}^{2}\eta_{10}}I_{15}+\frac{i_{R}^{2}\eta_{R}}{i_{2}^{2}\eta_{2}i_{11}^{2}\eta_{11}}I_{16}+\frac{i_{R}^{2}\eta_{R}}{i_{2}^{2}\eta_{2}i_{4}^{2}\eta_{4}}I_{9}+\frac{i_{R}^{2}\eta_{R}}{i_{2}^{2}\eta_{2}i_{4}^{2}\eta_{4}i_{8}^{2}\eta_{8}}I_{13}+\\ +\frac{i_{R}^{2}\eta_{R}}{i_{2}^{2}\eta_{2}i_{4}^{2}\eta_{4}i_{8}^{2}\eta_{8}i_{9}^{2}\eta_{9}}I_{14}+\frac{i_{R}^{2}\eta_{R}}{i_{2}^{2}\eta_{2}i_{4}^{2}\eta_{4}i_{5}^{2}\eta_{5}}I_{10}+\frac{i_{R}^{2}\eta_{R}}{i_{2}^{2}\eta_{2}i_{4}^{2}\eta_{4}i_{5}^{2}\eta_{5}i_{6}^{2}\eta_{6}}I_{11} \end{array}\right)\;\left[\mathrm{kg}\cdot m^{2}\right]$$

**Figure 22:**
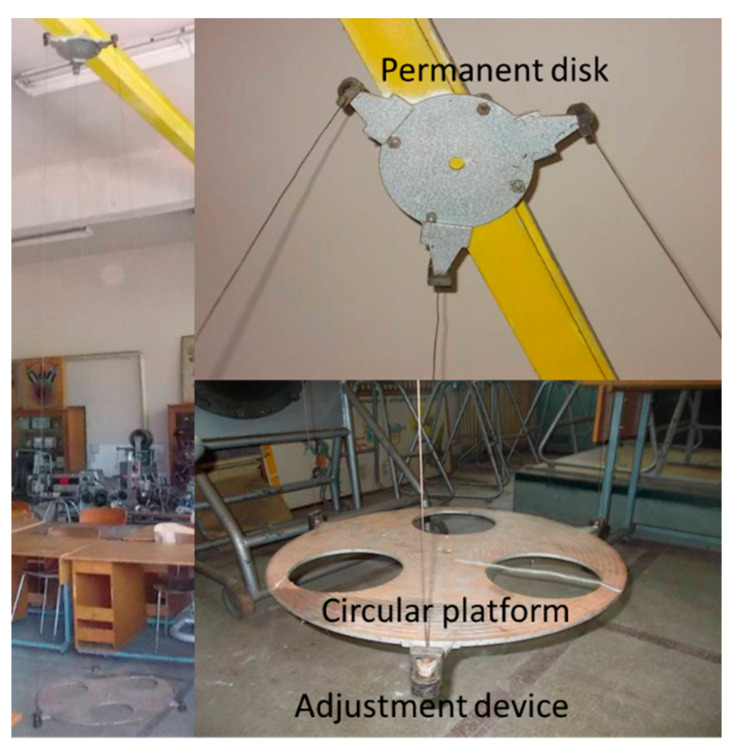
Three-wire pendulum.

The method involves the use of a three-wire pendulum. The three-wire pendulum is a device consisting of a movable circular platform, a fixed disk and three thin cables, characterized by the same diameter and the same length. The fixed disk is rigidly attached to a metal beam. The cables are used to support the circular platform at three equidistant points. The cables have a threaded adjustment device at their ends, which allows for obtaining parallelism between the mobile circular platform, the fixed disc and the floor (Figure 22). The technical data of the pendulum used are platform radius R = 0.345 m, fixed disk radius r = 0.18 m, wire length L = 3.22 m, platform mass mp = 13.7 kg.

**Figure 23:**
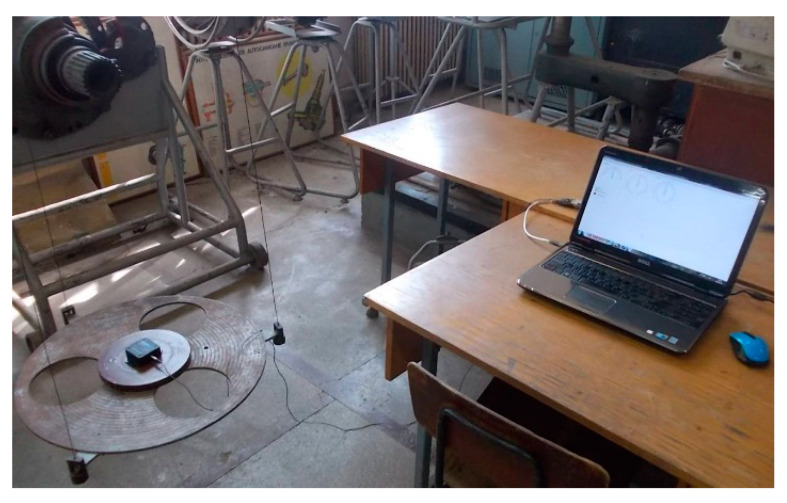
The main elements of the three-wire pendulum measuring chain.

To retrieve the necessary information to obtain the moments of inertia, in this case the oscillation period of the platform, the MicroStrain 3DM-GX1 inertial sensor is attached to the experimental device. The sensor is centered on the element subjected to the experimental process (Figure 23). The 3DM-GX1 Data Acquisition and Display Software sensor program is used to process the experimental data.

**Figure 24:**
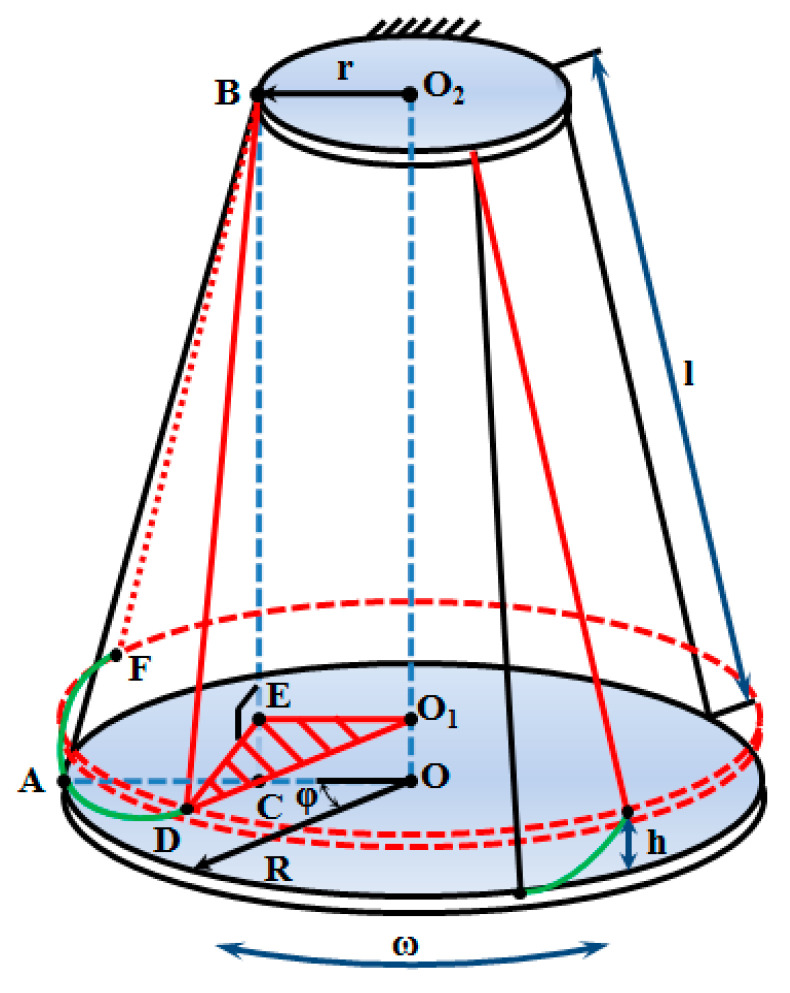
Schematic representation of the three-wire pendulum.

The experiment begins with the initial rotation of the platform at an angle of *φ* = 20…25°. This causes the platform to rise to a height of *h* (Figure 24).

By releasing the platform, it begins to perform circular oscillating movements, described by the relation:

(28) $$\varphi \left(t\right)=R\cdot \sin\left(\frac{2\pi }{T_{p}}\cdot t\right)\;\left[\mathrm{rad}\right]$$

The platform moves in a rotational movement around the OO_2_ axis and, at the same time, performs a translational movement along the OO_2_ axis of amplitude *h*. In this movement, the mobile platform is subject to the law of the conservation of energy. For this reason, it is known that at points *D* and *F* (points of maximum height), the kinetic energy of the platform is zero and the potential energy is maximum. During the movement from point *D* to point *A*, which represents the equilibrium position, the potential energy *E_p_* is transformed into kinetic energy *E_c_*. When passing through the equilibrium position, the kinetic energy is maximum. On the *A*–*F* path, kinetic energy is transformed into potential energy. The total energy of system *E* is constant and can be expressed as the sum of the kinetic energy and the potential energy of the studied system:

(29) {Ec=12I·ω2 [Nm]Ep=m·g·h [Nm]ω=dφdt=φ(t) [rads]|⇒E=Ec+Ep [Nm]

The height *h*, from a geometric point of view, is given by the length of the segment (OO_1_) and can be calculated as the difference between segment *BC* and segment *BE*.

(30) $$h=\frac{4\cdot r\cdot R\cdot \sin^{2}\left(\frac{\varphi }{2}\right)}{BC+BE}\;\left[m\right]$$

Given that angle *φ* varies in a very small range, it can be stated that, in terms of value, sin (*φ*) is approximately equal to the value of angle *φ* and that the length of segment *BC* is approximately equal to that of segment *BE*. Taking into account these hypotheses, the analytical expression of the maximum height (*h*) becomes:

(31) $$h=\frac{r\cdot R\cdot \varphi^{2}}{2\cdot l}\;\left[m\right]$$

By replacing the analytical expression of the maximum height in the potential energy definition relation, a function is obtained that depends on the square of the angular displacement of the circular platform with respect to the equilibrium position *φ*(*t*). Applying the law of the conservation of energy and performing the required simplification operations, the analytical expression of the moment of inertia is determined:

(32) Ep=m·g·r·R·φ(t)22·l [Nm]Ec=12·I·φ(t)2 [Nm]dEcdt+dEpdt=dEdt [Nms]φ(t)=R·sin(2πTp·t) [m]|→I=m·g·r·R4π2·l·Tp2 [kg·m2]

**Figure 25:**
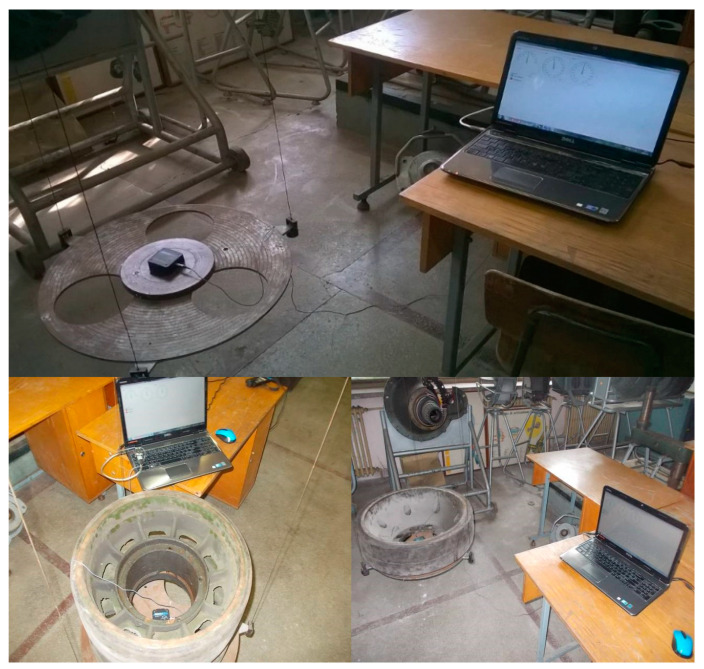
Representation of the experimental procedure for determining moments of inertia.

It can be seen that, in terms of value, the moment of inertia depends on the dimensional and mass characteristics of the experimental device *r*, *R*, *m* and the period of oscillation *T*. Determining the moments of inertia of the parts requires knowing the moment of inertia of the device, *i_p_*. Its value is subtracted from the moment of inertia of the assembly, consisting of the measuring device and part *I_p-i_* (Figure 25), resulting in the moment of inertia of the part (32).

In both situations, the calculation algorithm requires determining periods *T_p_* and *T_p-i_* specific to the oscillation movements of the mobile platform and the platform element *i* assembly. Both oscillation periods *T_p_* and *T_p-i_* are determined by applying the relations:

(33) $$T_{p}=\frac{t_{p}}{n}\;\left[s\right]\;T_{p-1}=\frac{t_{p-1}}{n}\;\left[s\right]$$

**Figure 26:**
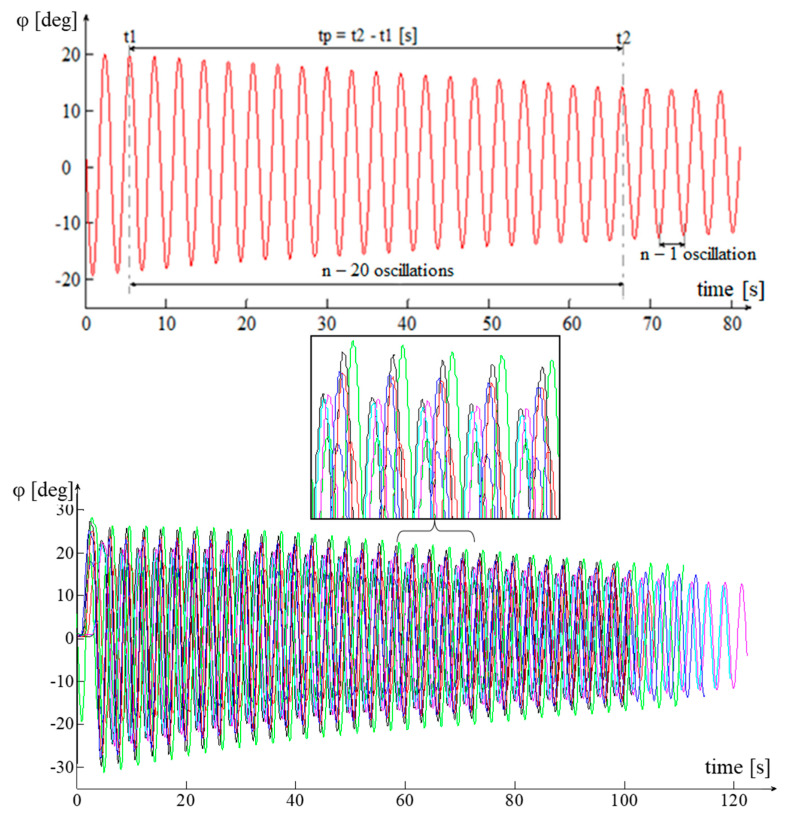
Graphical representation of the experimental data and the algorithm for extracting the data necessary to determine the period of oscillation, the red sample is their average, and each color represents the samples in Table 6.

In order to determine the oscillation times *t_p_*, *t_p-i_* and the number of oscillations *n* performed by the platform or the platform element *i* assembly, for the time periods *t_p_* or *t_p-i_*, the inertial sensor of the measuring chain is used—MicroStrain 3DM-GX1. For better accuracy of the results, the experimental process is repeated ten times, both for the platform and for the platform element *i* assembly. From the resulting data are extracted those necessary to determine the period of oscillation: oscillation time *t_p_* and oscillation time consumed by the element subjected to the measurement process *t_p-i_* for the execution of a fixed number *n* of oscillations—20 oscillations (Figure 26).

The oscillation periods *T_p_* and *T_p-i_* are calculated using the arithmetic mean of the oscillation periods specific to the ten experimental samples.

(34) $$T_{p}=\frac{1}{10}\cdot \sum_{j=1}^{10}T_{p}^{j}\;\left[s\right]\;T_{p-1}=\frac{1}{10}\cdot \sum_{j=1}^{10}T_{p-1}^{j}\;\left[s\right]$$

The oscillation periods are replaced in the relations of the moments of inertia specific to the platform *I_p_* and to the platform element *i* assemblies *I_p-i_* (Table 6).

(35) $$\{\begin{array}{l} I_{p}=\frac{m\cdot g\cdot r\cdot R}{4\pi^{2}\cdot l}\cdot T_{p}^{2}\;\left[\mathrm{kg}\cdot m^{2}\right]\\ I_{p-i}=\frac{m_{p-i}\cdot g\cdot r\cdot R}{4\pi^{2}\cdot l}\cdot T_{p-i}^{2}\;\left[\mathrm{kg}\cdot m^{2}\right] \end{array}$$

(36) $$I_{i}=I_{p-i}-I_{p}=\frac{g\cdot r\cdot R}{4\pi^{2}\cdot l}\cdot \left(m_{p-i}\cdot T_{p-i}^{2}-m\cdot T_{p}^{2}\right)\;\left[\mathrm{kg}\cdot m^{2}\right]$$

(37) $$\{\begin{array}{l} \delta_{i}=1+\frac{m_{ş}}{M_{a}}+\frac{I_{pr\_\exp}}{M_{a}\cdot r_{rm}^{2}}+\frac{{\left(I_{TR\_\exp}\right)}_{i}}{M_{a}\cdot r_{rm}^{2}}+\frac{{\left(i_{cd}^{2}\right)}_{i}\cdot {\left(\eta_{cd}\right)}_{i}}{M_{a}\cdot r_{rm}^{2}}\cdot I_{EP\_\exp}\cdot K_{h}\cdot \frac{d\omega_{p}}{d\omega_{t}}\;\left[-\right]\\ \delta_{IVm}=1+\frac{m_{ş}}{M_{a}}+\frac{I_{pr\_\exp}}{M_{a}\cdot r_{rm}^{2}}+\frac{{\left(i_{t}^{2}\right)}_{IV}\cdot {\left(\eta_{T}\right)}_{IV}}{M_{a}\cdot r_{rm}^{2}}\cdot {\left(I_{t}\right)}_{IVM}\;\left[-\right] \end{array}$$

**Figure 27:**
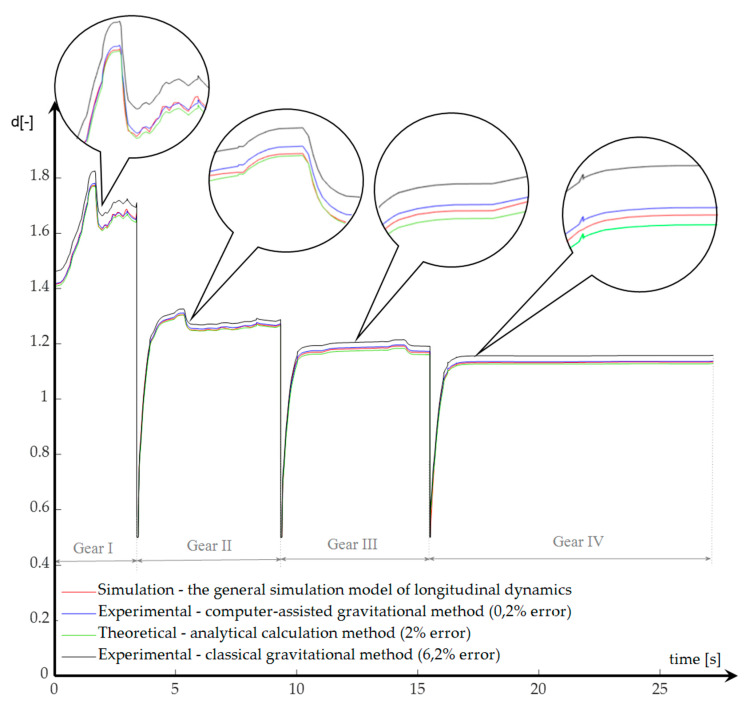
The variation of the coefficient of inertia masses resulting from the experimental data for starting the tracked vehicle.

The final relation for determining the variation of the coefficient of inertia masses contains data obtained both from the gravitational method and from the three-wire suspension method (Figure 27).

## 6. Discussion

The general virtual model for simulating the longitudinal dynamics of the vehicle required performing some tests. Two directions were followed: one aimed at determining the starting performances, and the other at obtaining the equivalent moments of inertia that entered the expression of approximation of the coefficient of inertia masses. To obtain the variation of the coefficient of inertia masses, three distinct methods were used: indirect, based on the signals generated from the interrogation of the virtual model; direct, which takes over the data resulting from the gravitational and three-wire pendulum tests; empirical, which is in fact an analytical–experimental method.

All these steps have led to the conclusion that this approach leads to smaller errors because there is no need to rely on the results of predictive models [31,32,33,34].

To date, the proposed solutions in this field have relied more on theoretical assumptions or computer simulations to demonstrate the effectiveness of virtual models for simulating the longitudinal dynamics of tracked heavy vehicles [32]. In addition, these vehicles can be deployed to perform tasks in unstructured environments, being forced to move and change position at speeds characterized by a strong start. On the other hand, different obstacles can appear, which slows the dynamics of the movement [33].

As the system is considered non-deformable, vibrations were not taken into account during the measurements. For this reason, we appreciate that no additional errors were obtained, which would vitiate the results.

Testing techniques will be further developed through the implementation of wireless technologies and artificial intelligence elements.

## 7. Conclusions

The results obtained by the computer-assisted gravitational method are more accurate than those obtained by the classical gravitational method. This is because the computer-assisted gravitational method uses two sets of data to determine moments of inertia: those on the acceleration section and those on the deceleration section. Compared to this, the classical gravitational method uses only the data on the acceleration section. It was pointed out that in the first three gears of the gearbox, the time dependence of the angular velocity was linear, both on the acceleration zone and on the start zone of the deceleration process. Instead, in the 4th gear and in the final part of the measurement process, due to the intensification of the friction phenomenon, the allure of the angular velocity variation curve became exponential towards the end of the acceleration or deceleration process. To approximate the specific values of acceleration and angular deceleration, the experimental samples were linearized (first-order polynomials were used as approximants). Linearization resulted in constant accelerations and decelerations. The least squares method approximated in good and very good limits the experimental data obtained by the computer-assisted gravitational method (with errors below 6%). From the comparison of the values of moments of inertia with those modeled in 3D (for the parts tested with the three-wire pendulum), it was observed that the differences between them were small. For the parts that could not be mounted on the three-wire device, the 3D models were used, with the veracity of the data being assumed by the precision of the 3D models.

## Figures and Tables

**Table 1:** Graph representing the evolution for turn commands.

		Execution Elements			
		A_SS_	A_SD_	A_RM_	A_Rm_
Progression	*Rectilinear*				
	*Turn right big radius*				
	*Turn right small radius*				
	*Turn left big radius*				
	*Turn left small radius*				
Reversing	*Rectilinear*				
	*Turn right big radius*				
	*Turn right small radius*				
	*Turn left big radius*				
	*Turn left small radius*				

**Table 2:** Experimental data obtained by applying the classical gravitational method.

CVP Gear	Sample Number	Mass (m_1_) (kg)	Mass (m_2_) (kg)	Time (t_1_) (s)	Time (t_2_) (s)
I gear	1a_gr1	500	---	160.0	---
	2a_gr1	500	---	160.4	---
	3a_gr1	500	---	161.1	---
	1b_gr1	---	600	---	34.90
	2b_gr1	---	600	---	34.90
	3b_gr1	---	600	---	34.65
II gear	1a_gr2	500	---	144.0	---
	2a_gr2	500	---	143.6	---
	3a_gr2	500	---	146.2	---
	1b_gr2	---	600	---	31.80
	2b_gr2	---	600	---	28.50
	3b_gr2	---	600	---	27.85
III gear	1a_gr3	500	---	124.0	---
	2a_gr3	500	---	114.4	---
	3a_gr3	500	---	118.7	---
	1b_gr3	---	600	---	24.05
	2b_gr3	---	600	---	24.35
	3b_gr3	---	600	---	22.15
IV gear	1a_gr4	500	---	7.460	---
	2a_gr4	500	---	7.798	---
	3a_gr4	500	---	7.929	---
	1b_gr4	---	600	---	4.1
	2b_gr4	---	600	---	3.6
	3b_gr4	---	600	---	3.9
Neutral	1a_neu	500	---	6.12	---
	2a_neu	500	---	6.78	---
	3a_neu	500	---	6.43	---
	1b_neu	---	600	---	3.12
	2b_neu	---	600	---	3.00
	3b_neu	---	600	---	3.20

**Table 3:** Equivalent reduced moment of inertia at the drive wheel determined using the classical gravitational method.

	Value of the Equivalent Moment of Inertia Reduced at the Drive Wheel				
	(kg·m^2^)				
Equivalent moment of inertia	I gear	II gear	III gear	IV gear	Neutral
I_echiv_rm_metoda_clasica_	8735	6508	4122	1046	678

**Table 4:** The results obtained from the process of deriving the experimental data on angular velocity.

Gearbox CVP	Weight Testing	Testing	Angular Acceleration (*ε*_1_)	Angular Deceleration (*ε*_2_)
	(kg)		(rad/s^2^)	(rad/s^2^)
First gear	m_2_–600	Test 1a_et1	0.0100	−0.2445
	m_2_–600	Test 2a_et1	0.0163	−0.1870
	m_2_–600	Test 3a_et1	0.0146	−0.1858
	m_2_–600	Test 4a_et1	0.0140	−0.1877
Second gear	m_2_–600	Test 1a_et2	0.0190	−0.2507
	m_2_–600	Test 2a_et2	0.0240	−0.2479
	m_2_–600	Test 3a_et2	0.0251	−0.2475
Third gear	m_2_–600	Test 1a_et3	0.0336	−0.3997
	m_2_–600	Test 2a_et3	0.0343	−0.4026
	m_2_–600	Test 3a_et3	0.0395	−0.3920
Fourth gear	m_2_–600	Test 1a_et4	1.1604	−0.6537
	m_2_–600	Test 2a_et4	1.1614	−0.6624
	m_2_–600	Test 3a_et4	1.2037	−0.6402
Neutral position	m_2_–600	Test 1a_etn	2.0304	−0.9135
	m_2_–600	Test 2a_etn	1.8918	−1.1062

**Table 5:** Equivalent moment of inertia reduced at the drive wheel determined by the computer-assisted gravitational method.

Equivalent Reduced Moment of Inertia Value at the Drive Wheel (kg·m^2^)									
First Gear		Second Gear		Third Gear		Fourth Gear		Neutral Position	
Testing	I_echiv	Testing	I_echiv	Testing	I_echiv	Testing	I_echiv	Testing	I_echiv
1a	7785.2	1a	5866.3	1a	3649.9	1a	846.33	1a	509.42
2a	7894.1	2a	5818.1	2a	3620.2	2a	841.77	2a	503.34
3a	7845.6	3a	5803	3a	3664.7	3a	831.70	3a	-----
I_echiv_as_calc_et1_		I_echiv_as_calc_et2_		I_echiv_as_calc_et3_		I_echiv_as_calc_et4_		I_echiv_as_calc_neutru_	
7841		5829		3645		840		506	

**Table 6:** Experimental data obtained with the three-wire pendulum method.

Tested Element	Sample Number	Period		Moment of Inertia *I_p_*
		Period	Period	or *I_p-i_* (kg·m^2^)
		*T_p_^j^* or *T_p-i_^j^* (s)	*T_p_* or *T_p-i_* (s)	
Three-wire pendulum platform	1	3.2530	3.2558	0.6959
	2	3.2420		
	3	3.2395		
	4	3.2630		
	5	3.2410		
	6	3.2752		
	7	3.2635		
	8	3.2575		
	9	3.2690		
	10	3.2540		
Platform—tensioning wheel assembly	1	3.0520	3.0532	5.9727
	2	3.0465		
	3	3.0560		
	4	3.0480		
	5	3.0490		
	6	3.0475		
	7	3.0605		
	8	3.0635	3.0532	5.9727
	9	3.0565		
	10	3.0520		

## Abbreviations

a [ms2]	longitudinal acceleration of the vehicle
$C_{cor}\;\left[-\right]$	linear correlation coefficient
$C_{\mathrm{cov}}\;\left[-\right]$	covariance coefficient
$E_{c}\;\left[\mathrm{Nm}\right]$	kinetic energy
$E_{p}\;\left[\mathrm{Nm}\right]$	potential energy
$f_{prs\alpha a}\;\left[-\right]$	the coefficient of the total advance resistance force
$F_{jA}\;\left[N\right]$	the inertial flow load factor of the vehicle
$F_{jpr}\;\left[N\right]$	the inertial forces of the propulsion system
$F_{j\underset{’}{s}}\;\left[N\right]$	the force of inertia of the track
$F_{p}\;\left[N\right]$	stationary propulsion force
${\tilde{F}}_{p}\;\left[N\right]$	dynamic propulsion force
$F_{t}\;\left[N\right]$	stationary traction force
${\tilde{F}}_{t}\;\left[N\right]$	dynamic traction force
g [ms2]	gravitational acceleration
$G_{a}\;\left[N\right]$	vehicle’s weight
HC	torque convertor
${\left(i_{cv}\right)}_{i}\;\left[-\right]$	the total kinematic transmission ratio in the gear (i = I … IV) of the planetary gearbox
$i_{cd}\;\left[-\right]$	the absolute transmission ratio of the mechanical transmission
$i_{dl}\;\left[-\right]$	the kinematic transmission ratio of the final drive
$i_{R}\;\left[-\right]$	the kinematic transmission ratio of the distribution mechanism
$i_{h}\;\left[-\right]$	torque convertor kinematic transmission ratio
$i_{h}^{\prime }\;\left[-\right]$	the inverse of the kinematic transmission ratio of the torque convertor
$i_{mî}\;\left[-\right]$	the kinematic transmission ratio of the planetary summation mechanism
${\left(i_{t}\right)}_{IV}$	the absolute transmission ratio for the operation of the transmission with the torque convertor blocked
$I_{0}\left[\mathrm{kg}\cdot m^{2}\right]$	the sum of the moments of inertia of the rotating elements with angular velocity $\omega_{b}$
$I_{1}\;\left[\mathrm{kg}\cdot m^{2}\right]$	the sum of the moments of inertia of the rotating elements with angular velocity $\omega_{a}$
$I_{2}\;\left[\mathrm{kg}\cdot m^{2}\right]$	the sum of the moments of inertia of the rotating elements with angular velocity $\omega_{2}$
$I_{3}\;\left[\mathrm{kg}\cdot m^{2}\right]$	the sum of the moments of inertia of the rotating elements with angular velocity $\omega_{5}$
$I_{4}\left[\mathrm{kg}\cdot m^{2}\right]$	the sum of the moments of inertia of the rotating elements with angular velocity $\omega_{8}$
$I_{21}\;\left[\mathrm{kg}\cdot m^{2}\right]$	the moment of inertia of the satellite plateaus of the planetary summation mechanisms
$I_{22}\;\left[\mathrm{kg}\cdot m^{2}\right]$	the moment of inertia of the satellite plateaus of the final drive
$I_{b4}\;\left[\mathrm{kg}\cdot m^{2}\right]$	equivalent moment of inertia reduced at the crankshaft
$I_{b5}\;\left[\mathrm{kg}\cdot m^{2}\right]$	equivalent moment of inertia reduced at the output shaft of the planetary gearbox
$I_{EP}/I_{EP\_\exp}\;\left[\mathrm{kg}\cdot m^{2}\right]$	equivalent moment of inertia reduced at the inlet shaft of the torque convertor pump
$I_{g}\;\left[\mathrm{kg}\cdot m^{2}\right]$	moments of inertia of the road wheel
$I_{ipr}\;\left[\mathrm{kg}\cdot m^{2}\right]$	the moments of inertia of the wheels contained in the tracked propulsion system
$I_{ji}\;\left[\mathrm{kg}\cdot m^{2}\right]$	equivalent reduced moments of inertia at the entry shaft of the planetary gearbox
$I_{mi}\;\left[\mathrm{kg}\cdot m^{2}\right]$	equivalent reduced moments of inertia at the output shaft of the reversing mechanism
$I_{pr}\;\left[\mathrm{kg}\cdot m^{2}\right]$	moment of inertia of the propulsion system wheels reduced to the drive sprocket
$I_{rî}\;\left[\mathrm{kg}\cdot m^{2}\right]$	moments of inertia of the tensioning wheel
$I_{rs}\;\left[\mathrm{kg}\cdot m^{2}\right]$	moments of inertia of the support roller
$I_{TR}\ldots I_{TR\_\exp}\;\left[\mathrm{kg}\cdot m^{2}\right]$	equivalent moment of inertia reduced to the drive sprocket axle
${\left(I_{t}\right)}_{IVm}\;\left[\mathrm{kg}\cdot m^{2}\right]$	equivalent moment of inertia reduced to the axle of the drive sprockets in mechanical mode
K [−]	torque convertor capacity factor
$K_{dl}\;\left[-\right]$	constant of the planetary mechanism of the final drive
$K_{h}\;\left[-\right]$	torque convertor transformation ratio
$K_{mî}\;\left[-\right]$	the constant of the planetary summation mechanism
*M_a_*	tank mass
$m_{c}\;\left[\mathrm{kg}\right]$	armored hull mass
$M_{fr}\;\left[\mathrm{Nm}\right]$	shear moment
$M_{Hp}\;\left[\mathrm{Nm}\right]$	the torque that loads the torque convertor pump shaft
$M_{Ht}\;\left[\mathrm{Nm}\right]$	the torque that loads the torque convertor turbine shaft
$M_{jpr}\;\left[\mathrm{Nm}\right]$	the torque reduced to the drive sprocket of the moments of inertial forces of the propulsion system wheels
$m_{pr}\;\left[\mathrm{kg}\right]$	propulsion system mass
$m_{\underset{’}{s}}\;\left[\mathrm{kg}\right]$	track mass
$M_{p}\;\left[\mathrm{Nm}\right]$	torque absorbed by torque convertor pump
$M_{rm}\;\left[\mathrm{Nm}\right]$	stationary drive sprocket torque
${\tilde{M}}_{rm}\;\left[\mathrm{Nm}\right]$	dynamic drive sprocket torque
n [−]	number of oscillations
n [−]	number of experimental values
np [rads]	the torque convertor pump
nt [rads]	angular speed of torque convertor turbine
r [m]	the radius of the drum on which the metal cable was wound
R [m]	amplitude of oscillating motion
$R_{a}\;\left[N\right]$	air resistance force
$r_{g}\;\left[m\right]$	road wheel radius
$R_{pr}\;\left[N\right]$	propulsion system resistance force
$R_{prsa\alpha }\;\left[N\right]$	total advance resistance force
$r_{rm}\;\left[m\right]$	drive sprocket radius
$r_{rî}\;\left[m\right]$	tensioning wheel radius
$r_{rs}\;\left[m\right]$	support roller radius
$R_{s}\;\left[N\right]$	runway resistance force
$R_{\alpha }\;\left[N\right]$	uphill resistance force
$R_{\delta }\;\left[N\right]$	acceleration resistance force
$s_{d}\;\left[m\right]$	starting distance
$S_{t}\;\left[-\right]$	standard deviations for the vector time t [s]
$S_{\omega }\;\left[-\right]$	standard deviations for the angular velocity ω [rads]
$t_{1}\;\left[s\right]$	times of lowering of mass weights $m_{1}\;\left[\mathrm{kg}\right]$ from height H [m]
$t_{2}\;\left[s\right]$	times of lowering of mass weights $m_{2}\;\left[\mathrm{kg}\right]$ from height H [m]
$t_{d}\;\left[s\right]$	starting time
$t_{p-i}\ldots t_{p}\;\left[s\right]$	the times elapsed during the *n* oscillations of the circular platform and the platform element *i* assembly
$T_{p}\;\left[s\right]$	the period of oscillation of the platform of the experimental device
$t_{s}\;\left[s\right]$	gear changing time
$\bar{t}\;\left[s\right]$	arithmetic means of experimental values - vectors t [s]
v [ms]…V [kmh]	vehicle speed
δ [−]	coefficient of inertia masses
$\delta_{m\_phc}\;\left[-\right]$	the coefficient of the inertial masses of rotation arranged upstream of the torque convertor
$\delta_{pr}\;\left[-\right]$	the coefficient of inertial masses of rotation of the tracked propulsion system
$\delta_{r}\;\left[-\right]$	the coefficient of inertial masses in rotational motion
$\delta_{t}\;\left[-\right]$	the coefficient of inertial masses in translational motion
$\delta_{thc\_rm}\;\left[-\right]$	the coefficient of the inertial masses of rotation arranged downstream of the torque convertor.
εp [rads2]	angular acceleration of torque convertor pump
εrm [rads2]	angular acceleration of drive sprocket
εt [rads2]	angular acceleration of torque convertor turbine
$\eta_{cd}\;\left[-\right]$	mechanical transmission efficiency
${\left(\eta_{cv}\right)}_{i}\;\left[-\right]$	the efficiency corresponding to the gear *(i)* of the planetary gearbox
$\eta_{di}\;\left[-\right]$	the efficiency of the planetary mechanism of the final drive
$\eta_{e}\;\left[-\right]$	efficiency of outer cylindrical
$\eta_{h}\;\left[-\right]$	torque convertor efficiency
$\eta_{i}\;\left[-\right]$	efficiency of inner cylindrical
$\eta_{k}\;\left[-\right]$	efficiency of bevel gears
$\eta_{mî}\;\left[-\right]$	the efficiency of the planetary summation mechanism and the lateral demultiplexer
$\eta_{mî-tf}\;\left[-\right]$	efficiency of summation mechanisms and final transmissions
$\eta_{R}\;\left[-\right]$	distribution mechanisms efficiency
${\left(\eta_{t}\right)}_{IV}\;\left[-\right]$	transmission efficiency with the torque convertor blocked
φ(t) [m]	elongation of the oscillating motion
ω [rads]	the angular velocity
ωa [rads]	the angular velocity of the input shafts of the gearbox
ωb [rads]	the angular velocity of the output shafts of the gearbox
ωe [rads]	angular speed at the crankshaft
ω^i [rads]	the value estimated by the approximation model
ωp [rads]	the torque convertor pump
ωrm [rads]	angular speed of the drive sprocket
ωt [rads]	angular speed of torque convertor turbine
ω¯ [rads]	arithmetic means of experimental values - vectors ω [rads]
